# Prevalence and influencing factors of vitamin D deficiency in women with polycystic ovary syndrome: a systematic review and meta-analysis

**DOI:** 10.3389/fnut.2026.1865564

**Published:** 2026-06-15

**Authors:** Manman Yao, Liya Ma, Xiao Li, Xuan Zhou, Dawei Zhang

**Affiliations:** 1 The Third Affiliated Hospital of Henan University of Chinese Medicine, Zhengzhou, China; 2 The Second Clinical Medical College of Henan University of Chinese Medicine, Zhengzhou, China; 3 Henan University of Chinese Medicine, Zhengzhou, China; 4 Henan Provincial Hospital of Traditional Chinese Medicine, Zhengzhou, China

**Keywords:** influencing factors, meta-analysis, polycystic ovary syndrome, prevalence, vitamin D deficiency

## Abstract

**Objective:**

To systematically evaluate the prevalence and influencing factors of vitamin D deficiency in women with polycystic ovary syndrome (PCOS), providing an evidence-based basis for vitamin D screening, nutritional intervention, and metabolic management in this population. Methods: Systematic searches were conducted in PubMed, Embase, Cochrane Library, Web of Science, and Scopus databases to identify studies on the prevalence and influencing factors of vitamin D deficiency in women with PCOS published from database inception until April 24, 2026. Two researchers independently performed literature screening, data extraction, and quality assessment. Meta-analysis was conducted using Stata 18.0 software.

**Results:**

Thirty studies involving 4,773 women with PCOS were included, of whom 2,745 had vitamin D deficiency. Meta-analysis demonstrated a pooled prevalence of vitamin D deficiency of 58% in women with PCOS, with substantial heterogeneity among studies (*I*^2^ = 97.06%, *p* < 0.001). Subgroup analysis indicated that the prevalence was higher in developing countries than in developed countries (65% vs. 46%) and higher in the Asian region than in the European region (67% vs. 48%). Although the difference in the BMI subgroup did not reach statistical significance, the obese subgroup exhibited a higher prevalence (68%) than the normal-weight subgroup (57%) and the overweight subgroup (55%). Meta-regression identified geographical region (*β* = 0.123, 95% CI = 0.042–0.204, *p* = 0.004) and national development level (*β* = 0.198, 95% CI = 0.057–0.340, *p* = 0.008) as the primary sources of heterogeneity. Sensitivity analysis and publication bias tests indicated that the results were stable, with no obvious publication bias. Influencing factors indicated that elevated BMI, increased WHR, insulin resistance, dyslipidemia, inflammatory status, hyperandrogenism, and relevant genetic polymorphisms may be associated with an increased risk of vitamin D deficiency, whereas higher HDL-C levels and regular outdoor exercise may exert protective effects.

**Conclusion:**

Women with PCOS demonstrate significantly higher prevalence of vitamin D deficiency, particularly prominent in populations from developing countries. Vitamin D deficiency may be closely associated with factors such as obesity, insulin resistance, chronic inflammation, and dyslipidemia. Clinical practice should prioritize screening 25(OH)D levels in women with PCOS. Combined with weight management, dietary optimization, outdoor activities, and necessary vitamin D supplementation, this may help improve nutritional status and metabolic risk management in the PCOS population.

**Systematic review registration:**

https://www.crd.york.ac.uk/PROSPERO/view/CRD420261377549, identifier CRD420261377549.

## 1 Introduction

Polycystic ovary syndrome (PCOS) is the most common endocrine-metabolic disorder among women of reproductive age. Characterized primarily by ovulatory dysfunction, hyperandrogenism, and polycystic ovarian morphology, its clinical manifestations include menstrual irregularities, infertility, hirsutism, and acne. PCOS is frequently comorbid with obesity, insulin resistance, dyslipidemia, and chronic low-grade inflammation, significantly impairing female reproductive health and long-term metabolic outcomes (1, 2). In recent years, the role of oxidative stress in the pathogenesis and progression of PCOS has garnered increasing attention (3). Oxidative stress is a pathological state resulting from an imbalance between excessive reactive oxygen species production and insufficient antioxidant defense capacity. It participates in the pathogenesis of endocrine disorders and metabolic abnormalities in PCOS by damaging ovarian granulosa cell function, disrupting normal follicular development, and exacerbating insulin resistance and inflammatory responses (4).

Vitamin D is a liposoluble steroid hormone with endocrine, immunomodulatory, and metabolic regulatory functions. Clinically, serum 25-hydroxyvitamin D (25(OH)D) levels are routinely measured to assess vitamin D status (5). Multiple studies indicate that vitamin D deficiency is prevalent among women with PCOS, and its occurrence correlates with diverse factors including obesity, insulin resistance, seasonal variations, dietary patterns, and lifestyle habits (6, 7). Vitamin D deficiency not only exacerbates insulin resistance and chronic inflammation but also reduces the body’s anti-oxidative capacity and elevates oxidative stress levels, further disrupting the ovarian microenvironment and the homeostasis of hormone synthesis and metabolism (8). Therefore, vitamin D supplementation holds significant importance for improving certain inflammatory markers, oxidative stress, and endocrine-metabolic parameters in patients with PCOS (9).

Current studies on the prevalence and influencing factors of vitamin D deficiency in women with PCOS exhibit substantial heterogeneity; the absence of a comprehensive overall assessment may relate to variations in study regions, sample sizes, PCOS diagnostic criteria, vitamin D detection methods, BMI levels, and other factors (10). Therefore, this study employed a systematic review and meta-analysis approach to quantitatively synthesize the overall prevalence of vitamin D deficiency in women with PCOS and further explore its primary influencing factors. This aims to provide an evidence-based basis for vitamin D screening, nutritional intervention, and comprehensive metabolic management in women with PCOS.

This study was conducted in accordance with the Preferred Reporting Items for Systematic Reviews and Meta-Analyses (PRISMA) reporting guidelines (11) and was preregistered in the International Prospective Register of Systematic Reviews (PROSPERO) platform (CRD420261377549).

## 2 Materials and methods

### 2.1 Inclusion criteria

(1) Study types included cohort studies, case–control studies, and cross-sectional studies; (2) the study subjects comprised female populations definitively diagnosed with polycystic ovary syndrome (PCOS), including adolescent and reproductive-aged women, with no restriction on ethnicity or race; (3) studies reported serum 25-hydroxyvitamin D [25(OH)D] levels and classified vitamin D status according to the clinical practice guideline *Evaluation, Treatment, and Prevention of Vitamin D Deficiency: An Endocrine Society Clinical Practice Guideline* published by the Endocrine Society (5), in which 25(OH)D < 20 ng/mL (<50 nmol/L) was defined as vitamin D deficiency, 20 ≤ 25(OH)D < 30 ng/mL as vitamin D insufficiency, and 25(OH)D ≥ 30 ng/mL as vitamin D sufficiency; studies employing thresholds of 25(OH)D ≤ 20 ng/mL or ≤50 nmol/L were considered essentially consistent with this definition; (4) the literature provided data enabling either direct acquisition or indirect calculation of vitamin D deficiency prevalence among women with PCOS; (5) studies reported influencing factors for vitamin D deficiency in women with PCOS, including PCOS phenotype, age, ethnicity/race, sun exposure or outdoor activity, dietary intake, BMI or fat distribution, glucose and lipid metabolism indicators, inflammatory or oxidative stress markers, and genotype-related phenotypic, biochemical, or genetic data, permitting direct extraction or indirect calculation of odds ratio (OR), relative risk (RR), or hazard ratio (HR) with corresponding 95% confidence intervals (95% CI); (6) publications were in English.

### 2.2 Exclusion criteria

(1) Studies from which data on the prevalence of vitamin D deficiency or its influencing factors in women with PCOS could not be extracted, or studies that reported vitamin D insufficiency alone without separately extractable data on vitamin D deficiency; (2) studies involving non-PCOS women, or literature in which PCOS data could not be isolated from other diseases/populations; (3) reviews, conference abstracts, case reports, letters, animal studies, basic experimental research, and interventional randomized controlled trials; (4) studies with low methodological quality, fundamentally flawed research design, or significant statistical errors; (5) duplicate publications or studies with overlapping data sources.

The target outcome of this study was vitamin D deficiency rather than vitamin D insufficiency. Because these two conditions are defined by different 25(OH)D thresholds, studies reporting vitamin D insufficiency alone without separately extractable deficiency data may lead to inconsistent outcome definitions and were therefore excluded. Interventions in randomized controlled trials may alter serum 25(OH)D levels and affect the assessment of natural prevalence and related factors; therefore, post-intervention data were excluded. If pre-intervention baseline data could be separately extracted and met the inclusion criteria, only baseline data were included.

### 2.3 Literature search strategy

Computerized searches of the PubMed, Embase, Cochrane Library, Web of Science, and Scopus databases were performed to identify literature reporting the prevalence or influencing factors of vitamin D deficiency in women with PCOS. The search covered records from database inception or the earliest searchable year in each database to April 24, 2026, with no additional restriction on the initial publication year. Literature searches employed a combination of subject headings and free-text terms, with adjustments tailored to each database’s specific features. References of included studies were also searched to supplement relevant data. The search strategy was based on core terms related to PCOS and vitamin D, while PCOS phenotypes, dietary intake, outdoor activity, obesity-related parameters, oxidative stress markers, and other influencing factors were specifically considered during full-text screening and data extraction. Taking PubMed as an example, the detailed search strategy is presented in Table 1.

**Table 1:** Search strategy for the PubMed database.

Search number	Search strategy
1	Polycystic Ovary Syndrome [Mesh] OR Ovary Syndrome, Polycystic [Title/Abstract] OR Syndrome, Polycystic Ovary [Title/Abstract] OR Polycystic Ovarian Syndrome [Title/Abstract] OR Ovarian Syndrome, Polycystic [Title/Abstract] OR Polycystic Ovaries [Title/Abstract] OR Sclerocystic Ovarian Degeneration [Title/Abstract] OR Ovarian Degeneration, Sclerocystic [Title/Abstract] OR Sclerocystic Ovary Syndrome [Title/Abstract] OR Stein-Leventhal Syndrome [Title/Abstract] OR Stein Leventhal Syndrome [Title/Abstract] OR Syndrome, Stein-Leventhal [Title/Abstract] OR Sclerocystic Ovaries [Title/Abstract] OR Ovary, Sclerocystic [Title/Abstract] OR Sclerocystic Ovary [Title/Abstract] OR PCOS [Title/Abstract]
2	Vitamin D [Mesh] OR Vitamin D [Title/Abstract] OR D, Vitamin [Title/Abstract] OR Vitamin D3 [Title/Abstract] OR D3, Vitamin [Title/Abstract] OR Vitamin D2 [Title/Abstract] OR D2, Vitamin [Title/Abstract] OR Cholecalciferol [Title/Abstract] OR Calciferol, Chole [Title/Abstract] OR Ergocalciferol [Title/Abstract] OR Calciferol, Ergo [Title/Abstract] OR 25-hydroxyvitamin D [Title/Abstract] OR Vitamin D, 25-Hydroxy [Title/Abstract] OR 25 Hydroxyvitamin D [Title/Abstract] OR 25(OH)D [Title/Abstract] OR 25 OH D [Title/Abstract] OR 25OHD [Title/Abstract] OR 1,25-dihydroxyvitamin D [Title/Abstract] OR Vitamin D, 1,25-Dihydroxy [Title/Abstract] OR 1,25(OH)2D [Title/Abstract] OR Calcitriol [Title/Abstract] OR Calcidiol [Title/Abstract] OR Hypovitaminosis D [Title/Abstract] OR Vitamin D Deficiency [Title/Abstract]
3	#1 AND #2

### 2.4 Literature screening and data extraction

Two researchers independently screened the literature, extracted data, and cross-checked their work. Initial screening involved reviewing titles and abstracts to exclude obviously irrelevant studies. Subsequently, full texts were reviewed to determine eligibility against the inclusion criteria. Disagreements were resolved through consultation with a third researcher. Extracted data included: first author, publication year, study region, study design, sample size, baseline characteristics of participants, number of affected individuals, and influencing factors. Authors were contacted via email to obtain supplementary data when necessary.

### 2.5 Quality assessment

Two researchers independently evaluated the risk of bias in the included studies and cross-verified the results. The Newcastle-Ottawa Scale (NOS) was used to assess the included cohort studies and case–control studies (12), with a maximum score of 9 points. Scores of 1–3 indicated low-quality literature, 4–6 indicated medium-quality literature, and 7–9 indicated high-quality literature. The included cross-sectional studies were evaluated using the Agency for Healthcare Research and Quality (AHRQ) bias risk assessment criteria for cross-sectional studies (13). The scale has a maximum score of 11 points, with 0–3 points indicating low-quality literature, 4–7 points indicating moderate-quality literature, and 8–11 points indicating high-quality literature.

### 2.6 Statistical methods

This study utilized Stata 18.0 software to conduct the meta-analysis. Prevalence was used as the primary effect size, with corresponding 95% confidence intervals (CIs) reported. Inter-study heterogeneity was assessed using the Q-test at a significance threshold of *α* = 0.1, complemented by the I^2^ statistic to quantify heterogeneity magnitude. When no significant heterogeneity existed among studies (*p* > 0.1 and I^2^ < 50%), a fixed-effects model was employed to pool effect sizes; if heterogeneity is present (*p* ≤ 0.1 and I^2^ ≥ 50%), first explore its sources through subgroup analysis and meta-regression; after excluding key clinical heterogeneity, conduct the meta-analysis using a random-effects model (14). When the number of included studies was ≥10, publication bias was assessed using funnel plots, Egger’s test, and Begg’s test. Where publication bias was present, the trim and fill method was employed to correct funnel plot asymmetry (15). Sensitivity analysis was performed using the sequential exclusion method: individual studies were removed one by one to observe changes in pooled results and assess the robustness of conclusions (16). Meta-analysis was conducted for influencing factors reported in over two included studies, employing OR with 95% CI as effect measures. The significance level for meta-analysis was *α* = 0.05.

## 3 Results

### 3.1 Literature search results

**Figure 1:**
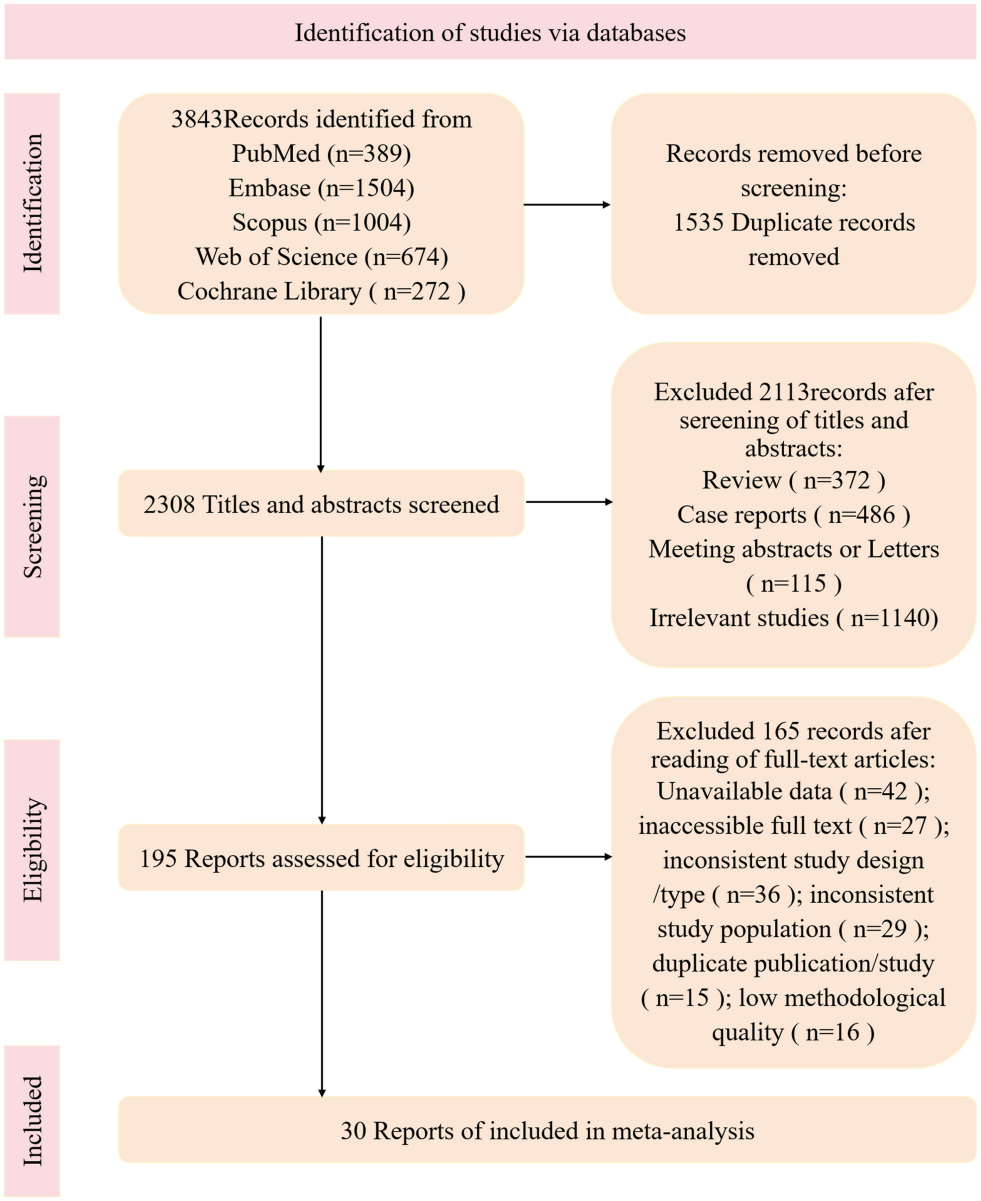
Flow diagram of literature screening.

Initial retrieval yielded 3,843 relevant articles: 389 from PubMed, 1,504 from Embase, 1,004 from Scopus, 674 from Web of Science, and 272 from Cochrane Library. Following duplicate removal, title/abstract screening, and full-text assessment, 30 articles were ultimately included (17–46). The literature screening process is presented in Figure 1 and Supplementary Table S1.

### 3.2 Basic characteristics and quality assessment results of included studies

This analysis included 30 studies, comprising a total of 4,773 women with PCOS, among whom 2,745 had vitamin D deficiency. Quality assessment identified 14 high-quality studies and 16 moderate-quality studies, indicating an overall good level of methodological quality among the included research. Detailed baseline characteristics of the included studies and quality assessment results are presented in Table 2 and Supplementary Tables S2–S4.

**Table 2:** Basic characteristics and quality assessment of the included studies.

Included studies	Study type	Country	Duration of the Study	Mean age (years)	Mean BMI (kg/m^2^)	Total sample size	Vitamin D deficiency cases	Prevalence rate	Diagnostic criteria for PCOS	25(OH)D measurement method	Influencing factors
Guo et al. (17)	Cohort study	China	2022–2024	30.63 ± 3.98	22.55 ± 3.43	111	27	24.30%	A	CLIA	—
Yari et al. (18)	Case–control study	Iran	2020	30.47 ± 6.51	—	66	45	68.20%	A	ELISA	—
Chakraborty et al. (19)	Case–control study	India	2023–2024	—	—	150	110	73.33%	A	CMIA	—
Akinola et al. (20)	Case–control study	Nigeria	—	26.90 ± 3.73	26.39 ± 4.34	60	20	33.30%	A	ELISA	—
Lejman-Larysz et al. (21)	Cross-sectional study	Poland	2018–2021	26.3 ± 5.1	27.26 ± 5.46	80	34	42.50%	A	CLIA	—
Nowak et al. (22)	Cross-sectional study	Poland	2021–2022	—	—	311	147	47.20%	B	CMIA	—
Rajbanshi et al. (23)	Cross-sectional study	Nepal	—	27.10 ± 4.09	24.81 ± 3.71	107	43	40.19%	A	E-CLIA	—
Shan et al. (24)	Cross-sectional study	China	2016–2020	26.05 ± 5.71	26.39 ± 5.84	625	539	86.24%	A	ECLIA	—
Bindayel (25)	Cross-sectional study	Saudi Arabia	2018	27 ± 5.3	29.4 ± 5.4	31	17	54.80%	A	ECLIA	—
Li et al. (26)	Cross-sectional study	China	2016–2019	27.1 ± 4.0	—	290	239	82.41%	A	LC–MS/MS	—
Wang et al. (27)	Cross-sectional study	China	2018–2019	28.4 ± 8.3	24.7 ± 6.5	169	92	54.40%	A	-	1,2,3,4,5,6,7,8,9
Davis et al. (28)	Case–control study	United States	2008–2012	—	—	137	29	21.20%	A	LC–MS/MS	—
Kensara (29)	Case–control study	Saudi Arabia	—	31.6 ± 6.4	22.2 ± 2.6	63	33	52.40%	A	ELISA	—
Krul-Poel et al. (30)	Cross-sectional study	Netherlands	—	34 ± 5	25.2	639	326	51.00%	A	LC–MS/MS	—
Mogili et al. (31)	Cross-sectional study	India	2016–2017	26.50 ± 3.98	27.9 ± 5.05	256	180	70.30%	A	CLIA	—
Kumar et al. (32)	Cross-sectional study	India	2015	28.6 ± 6.3	30.4 ± 6.1	100	44	44.00%	A	CLIA	—
Ng et al. (33)	Case–control study	Malaysia	2015–2016	29	30.4	40	37	92.50%	A	ECLIA	—
Ganie et al. (34)	Case–control study	India	2010–2011	23.35 ± 5.38	22.26 ± 2.17	122	107	87.70%	A	RIA	—
Scott et al. (35)	Cross-sectional study	Australia	—	28.43 ± 5.16	29.27 ± 8.07	40	29	72.50%	A	CLIA	—
Figurová et al. (36)	Case–control study	Slovakia	2010–2013	29.0 ± 5.0	—	99	37	37.00%	A	RIA	—
Moini et al. (37)	Cross-sectional study	Iran	2011–2012	28.2 ± 8.4	25.92 ± 4.71	125	115	92.00%	A	ELISA	—
Sadhir et al. (38)	Case–control study	United States	2008–2012	14.9	—	37	23	62.20%	C	LC–MS/MS	—
Kim et al. (39)	Case–control study	South Korea	—	34.1 ± 4.6	20.1 ± 3.0	38	22	57.90%	A	RIA	—
Bhattacharya and Jha (40)	Cross-sectional study	India	2010–2011	23.55 ± 4.42	26.25 ± 4.26	93	57	61.30%	A	—	10,11,12,13,14
Muscogiuri et al. (41)	Cross-sectional study	Italy	2011	26.0 ± 4.3	25.1 ± 5.10	38	14	37.00%	A	CLIA	—
Tsakova et al. (42)	Cross-sectional study	Bulgaria	—	24.31 ± 4.93	26.49 ± 7.50	70	44	62.90%	A	ECLIA	—
Li H. W. R. et al. (43)	Cross-sectional study	United Kingdom	2009	27.5	30.8	25	18	72.00%	A	LC–MS/MS	—
Wehr et al (44)	Cross-sectional study	Austria	2006–2010	27	24.2	545	170	31.20%	A	EIA	15,16
Wehr et al. (45)	Cross-sectional study	Austria	—	29 ± 7	26.2 ± 6.9	206	80	38.80%	A	ELISA	—
Yildizhan et al. (46)	Cross-sectional study	Turkey	2007–2008	26.01 ± 3.80	28.25 ± 6.76	100	67	67.00%	A	HPLC	—

A: Rotterdam 2003 criteria; B: Rotterdam 2018 criteria; C: National Institutes of Health criteria for polycystic ovary syndrome (NIH PCOS diagnostic criteria). CLIA, chemiluminescent immunoassay; ELISA, Enzyme-linked immunosorbent assay; CMIA, Chemiluminescent Microparticle Immunoassay; E-CLIA, Enhanced Chemiluminescent Immunoassay; ECLIA, Electrochemiluminescence Immunoassay; LC–MS/MS, Liquid Chromatography–Tandem Mass Spectrometry; RIA, Radioimmunoassay; EIA, Enzyme Immunoassay; HPLC, High performance liquid chromatography; 1: Body mass index (BMI) ≥ 25 kg/m^2^; 2: Waist-to-hip ratio (WHR) ≥ 0.85; 3: Fasting insulin ≥20 mIU/L; 4: Homeostatic model assessment-insulin resistance (HOMA-IR) ≥ 2.66; 5: Total cholesterol ≥5.69 mmol/L; 6: Low-density lipoprotein cholesterol (LDL-C) ≥ 3.1 mmol/L; 7: High-sensitivity C-reactive protein (hs-CRP) ≥ 3 mg/L; 8: High-density lipoprotein cholesterol (HDL-C) ≥ 1.55 mmol/L; 9: Daily outdoor exercise ≥1 time; 10. Elevated BMI (continuous); 11. Elevated Ferriman–Gallwey hyperandrogenism score (FG score); 12. Elevated total serum testosterone; 13. Elevated sex hormone-binding globulin (SHBG); 14. Elevated postprandial insulin; 15. Vitamin D binding protein gene GG genotype (GC GG genotype); 16. 7-Dehydrocholesterol Reductase gene GG genotype (DHCR7 GG genotype); “—” indicates that corresponding data were not described or reported in the included literature.

### 3.3 Meta-analysis results

#### 3.3.1 Overall prevalence

A meta-analysis of the prevalence of vitamin D deficiency in PCOS patients was conducted based on the 30 included studies. Given substantial heterogeneity among studies (*I*^2^ = 97.06%, *p* < 0.001), a random-effects model was employed to pool effect sizes. The pooled prevalence was 58% [95% CI = 49–66%], with detailed results presented in Table 3 and Supplementary Figure S1.

**Table 3:** Meta-analysis results of vitamin D deficiency prevalence in PCOS patients.

Subgroups	Number of included studies (articles)	Heterogeneity test		Effect model	Meta-analysis results	
		*I*^2^ (%)	*p*-value		Prevalence (95%CI)	*p*-value
Overall prevalence	30	97.06	<0.001	Random	58.0% (49.0%~66.0%)	
Study type						<0.001
Case–control study	10	95.86	<0.001	Random	60.0% (42.0%~76.0%)	
Cross-sectional study	19	97.48	<0.001	Random	59.0% (49.0%~69.0%)	
Cohort study	1	—	—	Random	24.0% (17.0%~33.0%)	
Region						<0.001
Asia	16	96.2	<0.001	Random	67.0% (56.0%~76.0%)	
Europe	10	90.67	<0.001	Random	48.0% (40.0%~56.0%)	
Americas	2	—	—	Random	29.0% (22.0%~36.0%)	
African	1	—	—	Random	33.0% (22.0%~47.0%)	
Oceania	1	—	—	Random	72.0% (56.0%~85.0%)	
National development status						0.002
Developed country	12	90.51	<0.001	Random	46.0% (38.0%~53.0%)	
Developing country	18	96.08	<0.001	Random	65.0% (55.0%~75.0%)	
Diagnostic Criteria for PCOS						0.051
Rotterdam 2003 criteria	28	97.22	<0.001	Random	58.0% (49.0%~67.0%)	
Rotterdam 2018 criteria	1	—	—	Random	47.0% (42.0%~53.0%)	
NIH PCOS diagnostic criteria	1	—	—	Random	62.0% (45.0%~78.0%)	
25(OH)D Measurement Method						0.728
CLIA-based	13	96.84	<0.001	Random	58.0% (45.0%~71.0%)	
EIA/ELISA	6	97.66	<0.001	Random	54.0% (32.0%~75.0%)	
RIA	3	—	—	Random	63.0% (26.0%~92.0%)	
LC–MS/MS	5	97.79	<0.001	Random	58.0% (35.0%~79.0%)	
HPLC	1	—	—	Random	67.0% (57.0%~76.0%)	
Mean age (years)						0.201
<25 years	4	89.3	<0.001	Random	70.0% (53.0%~84.0%)	
25–30 years	18	97.67	<0.001	Random	59.0% (47.0%~71.0%)	
>30 years	5	90.44	<0.001	Random	50.0% (36.0%~64.0%)	
Mean BMI (kg/m^2^)						0.459
Normal weight (BMI < 24)	4	97.32	<0.001	Random	57.0% (24.0%~86.0%)	
Overweight (24 ≤ BMI < 28)	13	98.05	<0.001	Random	55.0% (42.0%~68.0%)	
Obesity (BMI ≥ 28)	6	87.23	<0.001	Random	68.0% (52.0%~82.0%)	
Sample size (cases)						0.956
<100	14	84.13	<0.001	Random	58.0% (49.0%~67.0%)	
≥100	16	98.34	<0.001	Random	58.0% (46.0%~70.0%)	

“—” indicates that heterogeneity analysis was not performed for subgroups with fewer than 3 included studies. The CLIA-based subgroup covers CLIA, CMIA, E-CLIA and ECLIA assays.

#### 3.3.2 Subgroup analysis

Subgroup analyses of vitamin D deficiency prevalence in women with PCOS were conducted based on study design, geographic region, national development level, PCOS diagnostic criteria, vitamin D assay methodology, age, BMI category, and sample size. Results were as follows:

(1) By study design: pooled prevalence was 60% [95% CI = 42–76%] for case–control studies, 59% [95% CI = 49–69%] for cross-sectional studies, and 24% [95% CI = 17–33%] for cohort studies; (2) By region: Asian region at 67% [95% CI = 56–76%], European region at 48% [95% CI = 40–56%], American region at 29% [95% CI = 22–36%], African region at 33% [95% CI = 22–47%], Oceania region at 72% [95% CI = 56–85%]; (3) By national development level: developed countries at 46% [95% CI = 38–53%], developing countries at 65% [95% CI = 55–75%]; (4) By PCOS diagnostic criteria: studies using Rotterdam 2003 criteria reported 58% [95% CI = 49–67%], those using Rotterdam 2018 criteria reported 47% [95% CI = 42–53%], and those using NIH PCOS diagnostic criteria reported 62% [95% CI = 45–78%]; (5) Grouped by vitamin D detection method: studies using CLIA reported 58% [95% CI = 45–71%], EIA/ELISA 54% [95% CI = 32–75%], RIA 63% [95% CI = 26–92%], LC–MS/MS 58% [95% CI = 35–79%], and HPLC 67% [95% CI = 57–76%]; (6) Grouped by age: the <25 years group showed 70% [95% CI = 53–84%], the 25–30 years group 59% [95% CI = 47–71%], and the >30 years group 50% [95% CI = 36–64%]; (7) Grouped by BMI level: the normal weight (BMI < 24) group exhibited 57% [95% CI = 24–86%], the overweight (24 ≤ BMI < 28) group 55% [95% CI = 42–68%], and the obesity (BMI ≥ 28) group 68% [95% CI = 52–82%]; (8) Grouped by sample size: studies with sample sizes <100 showed 58% [95% CI = 49–67%], while studies with sample sizes ≥100 showed 58% [95% CI = 46–70%].

Between-group difference tests revealed statistically significant subgroup differences when stratified by study type, geographic region, and country development level (*p* < 0.05), whereas other subgroup comparisons showed no statistical significance (*p* ≥ 0.05). These subgroup analysis results appear in Table 3 and Supplementary Figures S2–S9.

### 3.4 Meta-regression analysis

This study conducted meta-regression analysis with study type, geographical region, national development level, PCOS diagnostic criteria, 25(OH)D detection method, mean age, mean BMI, and sample size as covariates to investigate sources of heterogeneity among the included studies. The results indicate that study region [*β* = 0.123, 95% CI = 0.042–0.204, *p* = 0.004] and national development level [β = 0.198, 95% CI = 0.057–0.340, *p* = 0.008] were significant factors influencing heterogeneity; no significant associations with heterogeneity were found for the remaining covariates (study type, PCOS diagnostic criteria, 25(OH)D detection method, mean age, mean BMI, and sample size) (*p* > 0.05). See Table 4 and Supplementary Figures S10–S17.

**Table 4:** Meta-regression results of vitamin D deficiency prevalence in PCOS patients.

Covariate	*β* (95% CI)	SE	*t*-value	*p*-value
Study type	−0.053 (−0.207 ~ 0.100)	0.075	−0.71	0.482
Region	0.123 (0.042 ~ 0.204)	0.040	3.11	0.004
National development status	0.198 (0.057 ~ 0.340)	0.069	2.87	0.008
Diagnostic criteria for PCOS	0.080 (−0.233 ~ 0.393)	0.153	0.52	0.604
25(OH)D Measurement method	0.029 (−0.054 ~ 0.112)	0.040	0.72	0.480
Mean age	−0.097 (−0.239 ~ 0.045)	0.069	−1.41	0.171
Mean BMI	0.056 (−0.093 ~ 0.206)	0.072	0.78	0.444
Sample size	0.004 (−0.160 ~ 0.168)	0.08	0.05	0.963

### 3.5 Sensitivity analysis

This study performed sensitivity analysis by sequentially excluding individual studies. Results demonstrated that after sequentially excluding any single study, the pooled prevalence ranged from 55.6 to 58.3%, exhibiting minimal fluctuation compared to the original pooled estimate. The 95% CIs following exclusion of each individual study overlapped with the original pooled 95% CI, indicating no substantial influence of any single study on the overall pooled effect. This supports the robustness of the findings. See Supplementary Figure S18.

### 3.6 Publication Bias test

The funnel plot indicated an approximately symmetrical distribution of study points, with no significant asymmetry observed. Further validation using the trim and fill method revealed no requirement for imputing additional studies. The pooled effect size remained unchanged before and after trimming, indicating minimal influence of publication bias on the combined results. Meanwhile, Egger’s linear regression test (*t* = −0.18, *p* = 0.856) and Begg’s rank correlation test (*z* = 0.37, *p* = 0.708) both indicated no risk of publication bias. Considering the above results, this study was minimally affected by publication bias, and the conclusions possess certain reliability. See Supplementary Figures S19–S20.

### 3.7 Assessment of influencing factors

Among the included studies, only three studies (27, 40, 44) reported relevant influencing factors for vitamin D deficiency in women with PCOS. Owing to variations across studies in methodological design, definitions of exposure factors, and formats for reporting effect sizes, coupled with the extremely limited number of studies included for any single influencing factor, a meta-analytic synthesis could not be performed for all included influencing factors. Obesity and adiposity distribution-related indicators included BMI ≥ 25 kg/m^2^, elevated BMI, and WHR ≥ 0.85; glucose metabolism and insulin resistance-related indicators included fasting insulin ≥20 mIU/L, HOMA-IR ≥ 2.66, and elevated postprandial insulin; lipid metabolism-related indicators included total cholesterol >5.69 mmol/L, LDL-C ≥ 3.1 mmol/L, and HDL-C ≥ 1.55 mmol/L, among which HDL-C ≥ 1.55 mmol/L was identified as a protective factor; inflammation/oxidative stress-related indicators mainly included hs-CRP ≥ 3 mg/L; hyperandrogenism-related indicators included elevated FG score, elevated total serum testosterone, and elevated SHBG; lifestyle-related factors mainly included outdoor exercise ≥1 time daily, which was identified as a protective factor; genetic factors included GC-GG genotype and DHCR7-GG genotype. It should be noted that BMI cannot fully reflect fat distribution and visceral fat accumulation. Therefore, WHR, waist circumference, body fat percentage, total fat content, visceral fat, and other obesity-related parameters were also considered in this study. However, the extractable effect estimates for obesity-related indicators in the included studies were mainly available for BMI and WHR, whereas visceral fat and total fat content were insufficiently reported; therefore, further quantitative analysis could not be performed. These results suggest that obesity and abnormal fat distribution, insulin resistance, dyslipidemia, inflammatory status, abnormal androgen levels, and genetic factors may be associated with an increased risk of vitamin D deficiency, whereas higher HDL-C levels and regular outdoor exercise may confer protective effects Table 5.

**Table 5:** Summary of influencing factors not included in the meta-analysis.

Influencing factors	OR (95% CI)	*p*-value	Influence on vitamin D deficiency
BMI ≥ 25 kg/m^2^ (27)	2.53 (1.23–5.18)	0.011	RF
WHR ≥ 0.85^[27]^	2.17 (1.17–3.92)	0.019	RF
Fasting insulin ≥20 mIU/L (27)	5.74 (1.96–38.90)	0.008	RF
HOMA-IR ≥ 2.66^[27]^	10.06 (2.58–73.20)	0.003	RF
Total cholesterol ≥5.69 mmol/L (27)	1.98 (0.93–2.86)	0.047	RF
LDL-C ≥ 3.1 mmol/L (27)	2.23 (1.88–7.43)	0.035	RF
hs-CRP ≥ 3 mg/L (27)	2.18 (1.85–6.37)	0.038	RF
HDL-C ≥ 1.55 mmol/L (27)	0.37 (0.25–0.89)	0.024	PF
Outdoor exercise ≥1 times daily (27)	0.49 (0.31–0.93)	0.032	PF
Elevated BMI (continuous) (40)	1.05 (1.01–1.22)	—	RF
Elevated FG score (40)	1.04 (1.03–1.16)	—	RF
Elevated total serum testosterone (40)	1.57 (1.09–3.57)	—	RF
Elevated SHBG (40)	1.02 (1.01–1.05)	—	RF
Elevated postprandial insulin (40)	2.00 (1.09–3.07)	—	RF
GC GG genotype (44)	2.53 (1.27–5.06)	0.009	RF
DHCR7 GG genotype (44)	2.66 (1.08–6.55)	0.033	RF

RF represents risk factor, and PF represents protective factor. Symbol “—” means that relevant *p*-values were not described in the original literature.

## 4 Discussion

This meta-analysis incorporated 30 studies involving 4,773 women with PCOS, demonstrating a pooled vitamin D deficiency prevalence of 58%, indicating this condition is highly prevalent within the PCOS population. PCOS is a disorder characterized by reproductive endocrine abnormalities and metabolic dysfunction, frequently accompanied by obesity, insulin resistance, dyslipidemia, and chronic low-grade inflammation (1, 47). Vitamin D not only regulates calcium and phosphorus metabolism but also modulates insulin signaling, suppresses inflammation, and enhances antioxidant defense mechanisms (48, 49). Consequently, the high prevalence of vitamin D deficiency in women with PCOS may represent not merely nutritional insufficiency, but rather a significant nutritional metabolic characteristic intrinsically linked to PCOS-associated metabolic abnormalities, inflammatory activation, and oxidative stress status.

Subgroup analysis demonstrated a significantly higher prevalence of vitamin D deficiency among women with PCOS in developing countries (65%) compared to developed countries (46%). Meta-regression analysis identified this difference as a primary source of heterogeneity. This disparity likely stems from the combined influence of multiple factors, including socioeconomic status, nutritional supply structures, and healthcare management systems. Previous studies indicate that vitamin D deficiency carries a higher disease burden in low and middle-income countries, where food fortification, supplement usage, and high-risk population screening constitute vital strategies for improving vitamin D status (50). Populations in developing countries may experience inadequate dietary vitamin D intake, suboptimal nutritional screening systems, low nutritional supplementation awareness, and insufficient health education dissemination; compounded by lifestyle factors including limited outdoor activity and significant obesity burden, collectively contributing to reduced 25(OH)D levels (51); Furthermore, pre-existing insulin resistance and chronic low-grade inflammation in PCOS patients exacerbate systemic oxidative stress imbalance. This imbalance potentially impairs vitamin D metabolism while simultaneously worsening insulin resistance and metabolic dysregulation, establishing a mutually reinforcing cycle of “vitamin D deficiency-oxidative stress activation-PCOS metabolic dysregulation” (8). By comparison, developed countries typically maintain relatively well-established nutrition and health management systems. The widespread availability of vitamin D-fortified foods, standardized clinical monitoring, and exogenous supplementation guidance help compensate for endogenous synthesis deficiencies, thereby sustaining adequate vitamin D levels (52), and may confer potential benefits to PCOS patients by ameliorating inflammatory responses, metabolic profiles, and oxidative stress status (9). This finding indicates that women with PCOS in developing countries constitute a high-risk group for vitamin D deficiency; nutritional screening, health education, and lifestyle improvements should be prioritized for this population.

Regional subgroup analysis revealed variations in vitamin D deficiency prevalence among women with PCOS across different regions. The prevalence was 67% in the Asian region compared to 48% in Europe. Meta-regression identified study region as a significant source of heterogeneity. However, it should be noted that the number of included studies from America, Africa, and Oceania was limited. Africa and Oceania each contributed only a single study; consequently, the stability of these results is constrained, and they should primarily serve as reference findings. Comparatively, the higher number of studies included from Asia and Europe lends greater explanatory value to the findings. Previous research indicates significant regional variations in vitamin D status and dietary intake levels globally, influenced by multiple factors including latitude, sun exposure, dietary patterns, food fortification, and supplement usage (51). The Asian region encompasses vast geographical areas with a large population base, and the included studies feature a high proportion of participants from developing countries. Certain populations may face challenges including inadequate dietary vitamin D intake, low prevalence of vitamin D-fortified foods, insufficient awareness of nutritional supplementation, and high-sugar high-fat diets (50), These factors readily promote obesity, insulin resistance, and chronic low-grade inflammation, consequently elevating oxidative stress levels and reducing vitamin D bioavailability (3). With its higher proportion of developed countries, Europe exhibits greater prevalence of food fortification, supplement usage, and nutritional health management. As food fortification has been demonstrated to reduce vitamin D deficiency risk, this may partially improve vitamin D intake and maintenance status (52).

Although subgroup analysis by BMI demonstrated no statistically significant differences, and meta-regression showed no significant impact on heterogeneity (*p* ≥ 0.05), a relatively clear clinical trend was observed. The prevalence of vitamin D deficiency among obese women with PCOS was 68%, higher than that in the normal-weight group (57%) and the overweight group (55%). This finding is largely consistent with the biological mechanisms through which obesity affects vitamin D status. Vitamin D, being a fat-soluble vitamin, is susceptible to sequestration or accumulation within adipose tissue upon its expansion, consequently reducing circulating 25(OH)D levels (53); Concurrently, obesity is frequently accompanied by insulin resistance and chronic low-grade inflammation, which may further affect vitamin D metabolism and bioavailability (54). However, the BMI subgroup differences in this study did not reach statistical significance, potentially attributable to the subgroup analysis employing study-level average BMI for stratification. Study-level covariate analysis cannot fully substitute individual-level association analysis, possibly introducing aggregation bias or ecological bias (55); Additionally, variations in study populations, geographic regions, dietary patterns, and detection methods across different studies may attenuate the statistical effects observed after BMI stratification. Therefore, the present findings do not negate the association between obesity and vitamin D deficiency. Integrating existing mechanistic and influencing factor evidence, obesity should still be considered a significant clinically relevant factor for vitamin D deficiency in women with PCOS, potentially contributing to its pathogenesis through mechanisms including fat accumulation, insulin resistance, inflammation activation, and oxidative stress imbalance (47).

From the perspective of potential mechanisms, obesity and central adiposity may reduce circulating 25(OH)D levels and its bioavailability through the storage or sequestration of fat-soluble vitamin D in adipose tissue (56, 57). Meanwhile, vitamin D may participate in insulin signaling regulation, inflammatory responses, and the maintenance of metabolic homeostasis through the vitamin D receptor (VDR) (48, 49). When vitamin D levels are insufficient, VDR-mediated metabolic regulation may be weakened, thereby being associated with PCOS-related pathological features such as insulin resistance, chronic low-grade inflammation, and hyperandrogenism (58, 59). Overall, vitamin D deficiency may be linked to metabolic and reproductive endocrine abnormalities in women with PCOS by affecting fat distribution, insulin sensitivity, inflammatory status, and endocrine-metabolic homeostasis.

Evaluation of influencing factors further supports the above conclusion. The primary reported risk factors included elevated BMI, increased WHR, elevated fasting insulin, elevated HOMA-IR, elevated total cholesterol, elevated LDL-C, and elevated hs-CRP; protective factors comprised elevated HDL-C and regular outdoor exercise. Additionally, elevated FG score, elevated total serum testosterone, elevated SHBG, elevated postprandial insulin, and genetic factors including GC-GG genotype and DHCR7-GG genotype were also associated with vitamin D deficiency in women with PCOS. Collectively, these factors predominantly cluster in domains encompassing obesity and abnormal fat distribution, insulin resistance, dyslipidemia, chronic inflammation, hyperandrogenism, and genetic susceptibility, indicating that vitamin D deficiency represents not an isolated nutritional inadequacy but is intrinsically linked to PCOS pathophysiological processes. Obesity and central adiposity reduce circulating 25(OH)D levels through fat-soluble vitamin sequestration effects (56) and promote insulin resistance and chronic low-grade inflammation; insulin resistance, hyperinsulinemia, and dyslipidemia are common metabolic abnormalities in women with PCOS and may be associated with reduced vitamin D levels (60); elevated hs-CRP indicates a state of chronic low-grade inflammation, which may also be related to vitamin D deficiency. Meanwhile, higher HDL-C levels may confer certain anti-inflammatory and lipid metabolism-protective effects (61). Regular outdoor exercise may exert protective effects by increasing sunlight exposure, promoting vitamin D synthesis, and improving insulin sensitivity.

Differences in genetic architecture may also affect susceptibility to vitamin D deficiency in women with PCOS. Evidence on influencing factors included in this study suggests that the GC-GG genotype and DHCR7-GG genotype are associated with an increased risk of vitamin D deficiency in women with PCOS (44). The GC gene encodes vitamin D-binding protein, which participates in the transport, storage, and bioavailability regulation of circulating 25(OH)D; GC-related SNPs or genotype differences may affect the level or function of vitamin D-binding protein, thereby leading to interindividual differences in 25(OH)D levels under similar sun exposure or intake conditions (62, 63). The DHCR7 gene encodes 7-dehydrocholesterol reductase, which participates in the conversion of 7-dehydrocholesterol to cholesterol. Because 7-dehydrocholesterol is an important precursor for cutaneous vitamin D3 synthesis, DHCR7-related genetic variants may alter 25(OH)D levels by affecting the availability of the substrate for vitamin D synthesis (64, 65). In addition, VDR-related polymorphisms may influence downstream vitamin D signaling and may be further associated with insulin sensitivity, androgen levels, and inflammatory responses (66). However, because most influencing factors were reported by single or only a few studies, and the definitions of variables and adjustment factors varied across studies, a meta-analysis could not be performed, and the relevant findings should be further validated by additional high-quality studies.

Additionally, in subgroup analyses stratified by study design, PCOS diagnostic criteria, 25(OH)D measurement methods, age, and sample size, prevalence fluctuated moderately across subgroups. However, most differences lacked statistical significance, and meta-regression demonstrated no significant impact on inter-study heterogeneity (*p* ≥ 0.05). When grouped by study design, prevalence rates in case–control and cross-sectional studies were relatively similar at 60 and 59% respectively, whereas cohort studies demonstrated a prevalence of 24%; although statistically significant between-group differences were observed, only one cohort study was included; its results are susceptible to influence from study design, follow-up setting, and sample selection bias. Consequently, this evidence does not suffice to establish study type itself as a factor influencing prevalence heterogeneity. Regarding PCOS diagnostic criteria, prevalence associated with Rotterdam 2003 criteria, Rotterdam 2018 criteria, and NIH PCOS diagnostic criteria was 58, 47, and 62%, respectively. Between-group differences approached but did not reach statistical significance. Furthermore, only single studies represented each of the latter two criteria, rendering the data insufficient to assess the impact of the diagnostic criteria themselves on prevalence. The between-group differences in the 25(OH)D assay methods, age subgroups, and sample size subgroups showed no statistical significance, indicating these factors were not primary sources of heterogeneity in the prevalence observed in this study. Notably, the <25-year age subgroup exhibited relatively higher prevalence, a trend potentially related to insufficient outdoor activity, unstable dietary patterns, and irregular lifestyles among younger populations (67, 68), but additional high-quality studies are required for further verification. The prevalence was 58% in studies with sample sizes <100 and ≥100, suggesting that the overall results of this study were not significantly influenced by sample size. Overall, while some prevalence fluctuations exist across subgroups, the core conclusion of a high vitamin D deficiency prevalence in women with PCOS remains consistent. Combined with sensitivity analysis and publication bias testing, the overall conclusions of this study demonstrate robust stability.

## 5 Strengths, limitations, and future direction

### 5.1 Strengths

This study systematically integrated data from 30 studies involving 4,773 women with PCOS and quantitatively synthesized the prevalence of vitamin D deficiency in women with PCOS, thereby providing a relatively comprehensive reflection of the overall burden of vitamin D deficiency in this population. Second, this study explored potential sources of heterogeneity through subgroup analysis and meta-regression across multiple dimensions, including study design, geographic region, national development level, PCOS diagnostic criteria, 25(OH)D measurement method, age, BMI level, and sample size, thereby improving the completeness of result interpretation. Finally, this study focused not only on the prevalence of vitamin D deficiency but also summarized influencing factors such as obesity, insulin resistance, dyslipidemia, inflammatory status, hyperandrogenism, lifestyle factors, and genetic factors, providing relatively systematic evidence for vitamin D screening and nutritional intervention in women with PCOS.

### 5.2 Limitations

This study still has several limitations: (1) Substantial heterogeneity existed among included studies. Subgroup analysis and meta-regression confirmed study region and national development level as primary heterogeneity sources; however, differences in study design, baseline population characteristics, lifestyle factors, and detection methods precluded complete elimination of heterogeneity’s potential impact on pooled results. (2) Several subgroups contained limited studies: cohort studies, Rotterdam 2018 criteria, NIH PCOS diagnostic criteria, HPLC detection methods, African region, and Oceania region each comprised only a single study, while the American region included only two studies. Consequently, the stability of these subgroup results is inadequate, providing suggestive value only. (3) The included studies consisted predominantly of cross-sectional and case–control designs. Although these can demonstrate correlations between vitamin D deficiency and metabolic abnormalities in PCOS, establishing causal relationships remains challenging. Thus, it is indeterminable whether vitamin D deficiency constitutes a cause or consequence of metabolic inflammation and oxidative stress abnormalities in PCOS. (4) Analysis of influencing factors: Only three studies reported extractable relevant factors. Variations in exposure definitions, adjustment factors, and effect size reporting across studies precluded quantitative synthesis, limiting further assessment of the effect strength of factors such as BMI, insulin resistance, dyslipidemia, inflammatory markers, and genetic polymorphism. (5) This study analyzed literature-level aggregate data. The lack of individual-level data, combined with insufficient reporting of key confounders such as PCOS phenotype, season, sunlight exposure duration, outdoor activity time, dietary vitamin D intake, medication use, and oxidative stress indicators in original studies, hindered an in-depth interpretation of the mechanisms underlying vitamin D deficiency.

### 5.3 Future direction

Future studies should adopt unified PCOS diagnostic criteria and vitamin D deficiency thresholds, and should standardize the reporting of key variables, including PCOS phenotype, age, ethnicity, season, sunlight exposure duration, outdoor activity, dietary vitamin D intake, medication use, inflammatory markers, and oxidative stress markers. Meanwhile, multicenter, large-sample, prospective studies are recommended, with enhanced reporting of SNP loci, rsIDs, and genotype distributions of vitamin D metabolism-related genes such as GC, DHCR7, and VDR, to further clarify the risk factors, causal relationships, and potential mechanisms of vitamin D deficiency in women with PCOS.

## 6 Summary

The findings indicate vitamin D deficiency is a prevalent nutritional and metabolic concern within the PCOS population. National development levels and geographic region represent significant sources of heterogeneity in prevalence estimates. Analysis of influencing factors suggests obesity, insulin resistance, dyslipidemia, inflammatory status, hyperandrogenemia, and genetic factors may correlate with vitamin D deficiency, whereas elevated HDL-C and regular outdoor exercise potentially exert protective effects. Clinical practice should prioritize 25(OH)D screening in women with PCOS, integrating weight management, dietary optimization, outdoor activity, and necessary vitamin D supplementation to provide a reference for improving nutritional status and metabolic risk management in this population.

## Data Availability

The original contributions presented in the study are included in the article/Supplementary material, further inquiries can be directed to the corresponding author.
