# Supplementary material for: Prevalence and influencing factors of vitamin D deficiency in women with polycystic ovary syndrome: a systematic review and meta-analysis
Source: Front Nutr. 2026 Jun 15;13:1865564. doi: 10.3389/fnut.2026.1865564 (PMC13311376; doi:10.3389/fnut.2026.1865564)

Supplementary Material

[1 Supplementary Table 1: Search Results 2](#_Toc228015045)

[2 Supplementary Table 2: Quality Assessment of Cohort Studies 3](#_Toc228015046)

[3 Supplementary Table 3: Quality Assessment of Cross-sectional Studies 3](#_Toc228015047)

[4 Supplementary Table 4: Quality Assessment of Case-control Studies 4](#_Toc228015048)

[5 Supplementary Figure 1: Forest plot of overall prevalence 6](#_Toc228015049)

[6 Supplementary Figure 2: Forest plot of subgroup analysis by study type 7](#_Toc228015050)

[7 Supplementary Figure 3: Forest plot of subgroup analysis by research region 8](#_Toc228015051)

[8 Supplementary Figure 4: Forest plot of subgroup analysis by national development status 9](#_Toc228015052)

[9 Supplementary Figure 5: Forest plot of subgroup analysis by diagnostic criteria for PCOS 10](#_Toc228015053)

[10 Supplementary Figure 6: Forest plot of subgroup analysis by 25(OH)D measurement method 11](#_Toc228015054)

[11 Supplementary Figure 7: Forest plot of subgroup analysis by mean age 12](#_Toc228015055)

[12 Supplementary Figure 8: Forest plot of subgroup analysis by mean BMI 13](#_Toc228015056)

[13 Supplementary Figure 9: Forest plot of subgroup analysis by sample size 14](#_Toc228015057)

[14 Supplementary Figure 10: Meta-regression plot by study type 15](#_Toc228015058)

[15 Supplementary Figure 11: Meta-regression plot by research region 15](#_Toc228015059)

[16 Supplementary Figure 12: Meta-regression plot by national development status 16](#_Toc228015060)

[17 Supplementary Figure 13: Meta-regression plot by diagnostic criteria for PCOS 16](#_Toc228015061)

[18 Supplementary Figure 14: Meta-regression plot by 25(OH)D measurement method 17](#_Toc228015062)

[19 Supplementary Figure 15: Meta-regression plot by mean age 17](#_Toc228015063)

[20 Supplementary Figure 16: Meta-regression plot by mean BMI 18](#_Toc228015064)

[21 Supplementary Figure 17: Meta-regression plot by sample size 18](#_Toc228015065)

[22 Supplementary Figure 18: Sensitivity analysis plot 19](#_Toc228015066)

[23 Supplementary Figure 19: Publication bias funnel plot 19](#_Toc228015067)

[24 Supplementary Figure 20: Funnel plot of trim-and-fill method 20](#_Toc228015068)

# Supplementary Table 1: Search Results

The search strategies for each database were implemented on April 24, 2026. After appropriate adaptation and adjustment, the strategies can also be applied to retrieve relevant studies in other electronic databases. PubMed (389)

| **Search number** | **Query** | **Search Details** | **Results** |
| --- | --- | --- | --- |
| **7** | ((Polycystic Ovary Syndrome [Mesh]) OR (Ovary Syndrome, Polycystic [Title/Abstract] OR Syndrome, Polycystic Ovary [Title/Abstract] OR Polycystic Ovarian Syndrome [Title/Abstract] OR Ovarian Syndrome, Polycystic [Title/Abstract] OR Polycystic Ovaries [Title/Abstract] OR Sclerocystic Ovarian Degeneration [Title/Abstract] OR Ovarian Degeneration, Sclerocystic [Title/Abstract] OR Sclerocystic Ovary Syndrome [Title/Abstract] OR Stein-Leventhal Syndrome [Title/Abstract] OR Stein Leventhal Syndrome [Title/Abstract] OR Syndrome, Stein-Leventhal [Title/Abstract] OR Sclerocystic Ovaries [Title/Abstract] OR Ovary, Sclerocystic [Title/Abstract] OR Sclerocystic Ovary [Title/Abstract] OR PCOS [Title/Abstract])) AND ((Vitamin D [Mesh]) OR (Vitamin D [Title/Abstract] OR D, Vitamin [Title/Abstract] OR Vitamin D3 [Title/Abstract] OR D3, Vitamin [Title/Abstract] OR Vitamin D2 [Title/Abstract] OR D2, Vitamin [Title/Abstract] OR Cholecalciferol [Title/Abstract] OR Calciferol, Chole [Title/Abstract] OR Ergocalciferol [Title/Abstract] OR Calciferol, Ergo [Title/Abstract] OR 25-hydroxyvitamin D [Title/Abstract] OR Vitamin D, 25-Hydroxy [Title/Abstract] OR 25 Hydroxyvitamin D [Title/Abstract] OR 25(OH)D [Title/Abstract] OR 25 OH D [Title/Abstract] OR 25OHD [Title/Abstract] OR 1,25-dihydroxyvitamin D [Title/Abstract] OR Vitamin D, 1,25-Dihydroxy [Title/Abstract] OR 1,25(OH)2D [Title/Abstract] OR Calcitriol [Title/Abstract] OR Calcidiol [Title/Abstract] OR Hypovitaminosis D [Title/Abstract] OR Vitamin D Deficiency [Title/Abstract])) | ("polycystic ovary syndrome"[MeSH Terms] OR ("ovary syndrome polycystic"[Title/Abstract] OR "syndrome polycystic ovary"[Title/Abstract] OR "polycystic ovarian syndrome"[Title/Abstract] OR "ovarian syndrome polycystic"[Title/Abstract] OR "polycystic ovaries"[Title/Abstract] OR "sclerocystic ovarian degeneration"[Title/Abstract] OR ((("Ovarian"[All Fields] OR "ovarians"[All Fields]) AND ("degenerate"[All Fields] OR "degenerated"[All Fields] OR "degenerately"[All Fields] OR "degenerates"[All Fields] OR "degenerating"[All Fields] OR "Degeneration"[All Fields] OR "degenerations"[All Fields])) AND "Sclerocystic"[Title/Abstract]) OR "sclerocystic ovary syndrome"[Title/Abstract] OR "stein leventhal syndrome"[Title/Abstract] OR "stein leventhal syndrome"[Title/Abstract] OR "syndrome stein leventhal"[Title/Abstract] OR "sclerocystic ovaries"[Title/Abstract] OR (("ovarial"[All Fields] OR "Ovary"[MeSH Terms] OR "Ovary"[All Fields] OR "Ovaries"[All Fields] OR "ovary s"[All Fields]) AND "Sclerocystic"[Title/Abstract]) OR "sclerocystic ovary"[Title/Abstract] OR "PCOS"[Title/Abstract])) AND ("vitamin d"[MeSH Terms] OR "ergocalciferols"[MeSH Terms] OR ((((((("vitamin d"[Title/Abstract] OR "d vitamin"[Title/Abstract] OR "vitamin d3"[Title/Abstract] OR "d3 vitamin"[Title/Abstract] OR "vitamin d2"[Title/Abstract] OR "d2 vitamin"[Title/Abstract] OR "Cholecalciferol"[Title/Abstract] OR (("ergocalciferols"[Supplementary Concept] OR "ergocalciferols"[All Fields] OR "calciferol"[All Fields] OR "ergocalciferols"[MeSH Terms] OR "calciferols"[All Fields]) AND "Chole"[Title/Abstract]) OR "Ergocalciferol"[Title/Abstract] OR (("ergocalciferols"[Supplementary Concept] OR "ergocalciferols"[All Fields] OR "calciferol"[All Fields] OR "ergocalciferols"[MeSH Terms] OR "calciferols"[All Fields]) AND "Ergo"[Title/Abstract]) OR "25 hydroxyvitamin d"[Title/Abstract] OR "vitamin d 25 hydroxy"[Title/Abstract] OR "25 hydroxyvitamin d"[Title/Abstract] OR 25[UID]) AND ("hydroxide ion"[Supplementary Concept] OR "hydroxide ion"[All Fields] OR "OH"[All Fields])) AND "D"[Title/Abstract]) OR "25 oh d"[Title/Abstract] OR "25OHD"[Title/Abstract] OR "1 25 dihydroxyvitamin d"[Title/Abstract] OR "vitamin d 1 25 dihydroxy"[Title/Abstract] OR 1,25[UID]) AND ("hydroxide ion"[Supplementary Concept] OR "hydroxide ion"[All Fields] OR "OH"[All Fields])) AND "2D"[Title/Abstract]) OR "Calcitriol"[Title/Abstract] OR "Calcidiol"[Title/Abstract] OR "hypovitaminosis d"[Title/Abstract] OR "vitamin d deficiency"[Title/Abstract])) | 389 |
| **6** | (Vitamin D [Mesh]) OR (Vitamin D [Title/Abstract] OR D, Vitamin [Title/Abstract] OR Vitamin D3 [Title/Abstract] OR D3, Vitamin [Title/Abstract] OR Vitamin D2 [Title/Abstract] OR D2, Vitamin [Title/Abstract] OR Cholecalciferol [Title/Abstract] OR Calciferol, Chole [Title/Abstract] OR Ergocalciferol [Title/Abstract] OR Calciferol, Ergo [Title/Abstract] OR 25-hydroxyvitamin D [Title/Abstract] OR Vitamin D, 25-Hydroxy [Title/Abstract] OR 25 Hydroxyvitamin D [Title/Abstract] OR 25(OH)D [Title/Abstract] OR 25 OH D [Title/Abstract] OR 25OHD [Title/Abstract] OR 1,25-dihydroxyvitamin D [Title/Abstract] OR Vitamin D, 1,25-Dihydroxy [Title/Abstract] OR 1,25(OH)2D [Title/Abstract] OR Calcitriol [Title/Abstract] OR Calcidiol [Title/Abstract] OR Hypovitaminosis D [Title/Abstract] OR Vitamin D Deficiency [Title/Abstract]) | "vitamin d"[MeSH Terms] OR "ergocalciferols"[MeSH Terms] OR ((((((("vitamin d"[Title/Abstract] OR "d vitamin"[Title/Abstract] OR "vitamin d3"[Title/Abstract] OR "d3 vitamin"[Title/Abstract] OR "vitamin d2"[Title/Abstract] OR "d2 vitamin"[Title/Abstract] OR "Cholecalciferol"[Title/Abstract] OR (("ergocalciferols"[Supplementary Concept] OR "ergocalciferols"[All Fields] OR "calciferol"[All Fields] OR "ergocalciferols"[MeSH Terms] OR "calciferols"[All Fields]) AND "Chole"[Title/Abstract]) OR "Ergocalciferol"[Title/Abstract] OR (("ergocalciferols"[Supplementary Concept] OR "ergocalciferols"[All Fields] OR "calciferol"[All Fields] OR "ergocalciferols"[MeSH Terms] OR "calciferols"[All Fields]) AND "Ergo"[Title/Abstract]) OR "25 hydroxyvitamin d"[Title/Abstract] OR "vitamin d 25 hydroxy"[Title/Abstract] OR "25 hydroxyvitamin d"[Title/Abstract] OR 25[UID]) AND ("hydroxide ion"[Supplementary Concept] OR "hydroxide ion"[All Fields] OR "OH"[All Fields])) AND "D"[Title/Abstract]) OR "25 oh d"[Title/Abstract] OR "25OHD"[Title/Abstract] OR "1 25 dihydroxyvitamin d"[Title/Abstract] OR "vitamin d 1 25 dihydroxy"[Title/Abstract] OR 1,25[UID]) AND ("hydroxide ion"[Supplementary Concept] OR "hydroxide ion"[All Fields] OR "OH"[All Fields])) AND "2D"[Title/Abstract]) OR "Calcitriol"[Title/Abstract] OR "Calcidiol"[Title/Abstract] OR "hypovitaminosis d"[Title/Abstract] OR "vitamin d deficiency"[Title/Abstract]) | 84,834 |
| **5** | Vitamin D [Title/Abstract] OR D, Vitamin [Title/Abstract] OR Vitamin D3 [Title/Abstract] OR D3, Vitamin [Title/Abstract] OR Vitamin D2 [Title/Abstract] OR D2, Vitamin [Title/Abstract] OR Cholecalciferol [Title/Abstract] OR Calciferol, Chole [Title/Abstract] OR Ergocalciferol [Title/Abstract] OR Calciferol, Ergo [Title/Abstract] OR 25-hydroxyvitamin D [Title/Abstract] OR Vitamin D, 25-Hydroxy [Title/Abstract] OR 25 Hydroxyvitamin D [Title/Abstract] OR 25(OH)D [Title/Abstract] OR 25 OH D [Title/Abstract] OR 25OHD [Title/Abstract] OR 1,25-dihydroxyvitamin D [Title/Abstract] OR Vitamin D, 1,25-Dihydroxy [Title/Abstract] OR 1,25(OH)2D [Title/Abstract] OR Calcitriol [Title/Abstract] OR Calcidiol [Title/Abstract] OR Hypovitaminosis D [Title/Abstract] OR Vitamin D Deficiency [Title/Abstract] | (((((("vitamin d"[Title/Abstract] OR "d vitamin"[Title/Abstract] OR "vitamin d3"[Title/Abstract] OR "d3 vitamin"[Title/Abstract] OR "vitamin d2"[Title/Abstract] OR "d2 vitamin"[Title/Abstract] OR "Cholecalciferol"[Title/Abstract] OR (("ergocalciferols"[Supplementary Concept] OR "ergocalciferols"[All Fields] OR "calciferol"[All Fields] OR "ergocalciferols"[MeSH Terms] OR "calciferols"[All Fields]) AND "Chole"[Title/Abstract]) OR "Ergocalciferol"[Title/Abstract] OR (("ergocalciferols"[Supplementary Concept] OR "ergocalciferols"[All Fields] OR "calciferol"[All Fields] OR "ergocalciferols"[MeSH Terms] OR "calciferols"[All Fields]) AND "Ergo"[Title/Abstract]) OR "25 hydroxyvitamin d"[Title/Abstract] OR "vitamin d 25 hydroxy"[Title/Abstract] OR "25 hydroxyvitamin d"[Title/Abstract] OR 25[UID]) AND ("hydroxide ion"[Supplementary Concept] OR "hydroxide ion"[All Fields] OR "OH"[All Fields])) AND "D"[Title/Abstract]) OR "25 oh d"[Title/Abstract] OR "25OHD"[Title/Abstract] OR "1 25 dihydroxyvitamin d"[Title/Abstract] OR "vitamin d 1 25 dihydroxy"[Title/Abstract] OR 1,25[UID]) AND ("hydroxide ion"[Supplementary Concept] OR "hydroxide ion"[All Fields] OR "OH"[All Fields])) AND "2D"[Title/Abstract]) OR "Calcitriol"[Title/Abstract] OR "Calcidiol"[Title/Abstract] OR "hypovitaminosis d"[Title/Abstract] OR "vitamin d deficiency"[Title/Abstract] | 28,948 |
| **4** | Vitamin D [Mesh] | "vitamin d"[MeSH Terms] OR "ergocalciferols"[MeSH Terms] | 74,297 |
| **3** | (Polycystic Ovary Syndrome [Mesh]) OR (Ovary Syndrome, Polycystic [Title/Abstract] OR Syndrome, Polycystic Ovary [Title/Abstract] OR Polycystic Ovarian Syndrome [Title/Abstract] OR Ovarian Syndrome, Polycystic [Title/Abstract] OR Polycystic Ovaries [Title/Abstract] OR Sclerocystic Ovarian Degeneration [Title/Abstract] OR Ovarian Degeneration, Sclerocystic [Title/Abstract] OR Sclerocystic Ovary Syndrome [Title/Abstract] OR Stein-Leventhal Syndrome [Title/Abstract] OR Stein Leventhal Syndrome [Title/Abstract] OR Syndrome, Stein-Leventhal [Title/Abstract] OR Sclerocystic Ovaries [Title/Abstract] OR Ovary, Sclerocystic [Title/Abstract] OR Sclerocystic Ovary [Title/Abstract] OR PCOS [Title/Abstract]) | "polycystic ovary syndrome"[MeSH Terms] OR ("ovary syndrome polycystic"[Title/Abstract] OR "syndrome polycystic ovary"[Title/Abstract] OR "polycystic ovarian syndrome"[Title/Abstract] OR "ovarian syndrome polycystic"[Title/Abstract] OR "polycystic ovaries"[Title/Abstract] OR "sclerocystic ovarian degeneration"[Title/Abstract] OR ((("Ovarian"[All Fields] OR "ovarians"[All Fields]) AND ("degenerate"[All Fields] OR "degenerated"[All Fields] OR "degenerately"[All Fields] OR "degenerates"[All Fields] OR "degenerating"[All Fields] OR "Degeneration"[All Fields] OR "degenerations"[All Fields])) AND "Sclerocystic"[Title/Abstract]) OR "sclerocystic ovary syndrome"[Title/Abstract] OR "stein leventhal syndrome"[Title/Abstract] OR "stein leventhal syndrome"[Title/Abstract] OR "syndrome stein leventhal"[Title/Abstract] OR "sclerocystic ovaries"[Title/Abstract] OR (("ovarial"[All Fields] OR "Ovary"[MeSH Terms] OR "Ovary"[All Fields] OR "Ovaries"[All Fields] OR "ovary s"[All Fields]) AND "Sclerocystic"[Title/Abstract]) OR "sclerocystic ovary"[Title/Abstract] OR "PCOS"[Title/Abstract]) | 27,987 |
| **2** | Ovary Syndrome, Polycystic [Title/Abstract] OR Syndrome, Polycystic Ovary [Title/Abstract] OR Polycystic Ovarian Syndrome [Title/Abstract] OR Ovarian Syndrome, Polycystic [Title/Abstract] OR Polycystic Ovaries [Title/Abstract] OR Sclerocystic Ovarian Degeneration [Title/Abstract] OR Ovarian Degeneration, Sclerocystic [Title/Abstract] OR Sclerocystic Ovary Syndrome [Title/Abstract] OR Stein-Leventhal Syndrome [Title/Abstract] OR Stein Leventhal Syndrome [Title/Abstract] OR Syndrome, Stein-Leventhal [Title/Abstract] OR Sclerocystic Ovaries [Title/Abstract] OR Ovary, Sclerocystic [Title/Abstract] OR Sclerocystic Ovary [Title/Abstract] OR PCOS [Title/Abstract] | "ovary syndrome polycystic"[Title/Abstract] OR "syndrome polycystic ovary"[Title/Abstract] OR "polycystic ovarian syndrome"[Title/Abstract] OR "ovarian syndrome polycystic"[Title/Abstract] OR "polycystic ovaries"[Title/Abstract] OR "sclerocystic ovarian degeneration"[Title/Abstract] OR ((("Ovarian"[All Fields] OR "ovarians"[All Fields]) AND ("degenerate"[All Fields] OR "degenerated"[All Fields] OR "degenerately"[All Fields] OR "degenerates"[All Fields] OR "degenerating"[All Fields] OR "Degeneration"[All Fields] OR "degenerations"[All Fields])) AND "Sclerocystic"[Title/Abstract]) OR "sclerocystic ovary syndrome"[Title/Abstract] OR "stein leventhal syndrome"[Title/Abstract] OR "stein leventhal syndrome"[Title/Abstract] OR "syndrome stein leventhal"[Title/Abstract] OR "sclerocystic ovaries"[Title/Abstract] OR (("ovarial"[All Fields] OR "Ovary"[MeSH Terms] OR "Ovary"[All Fields] OR "Ovaries"[All Fields] OR "ovary s"[All Fields]) AND "Sclerocystic"[Title/Abstract]) OR "sclerocystic ovary"[Title/Abstract] OR "PCOS"[Title/Abstract] | 23,582 |
| **1** | Polycystic Ovary Syndrome [Mesh] | "polycystic ovary syndrome"[MeSH Terms] | 21,174 |

# Supplementary Table 2: Quality Assessment of Cohort Studies

| **Included Studies** | **Selection** | | | | **Comparability** | **Exposure** | | | **Total Score** |
| --- | --- | --- | --- | --- | --- | --- | --- | --- | --- |
|  | **Representativeness of Exposed Cohort** | **Selection of Unexposed Cohort** | **Ascertainment of Exposure** | **No Outcome Events in Participants at Study Onset** | **Comparability of Cohorts Based on Design or Analysis** | **Outcome Assessment** | **Adequacy of Follow-up** | **Completeness of Follow-up** |  |
| Guo L, 2025 | 1 | 1 | 1 | 1 | 2 | 1 | 1 | 1 | 9 |

# Supplementary Table 3: Quality Assessment of Cross-sectional Studies

| **Included Studies** | **①** | **②** | **③** | **④** | **⑤** | **⑥** | **⑦** | **⑧** | **⑨** | **⑩** | **⑪** | **Total Score** |
| --- | --- | --- | --- | --- | --- | --- | --- | --- | --- | --- | --- | --- |
| Lejman-Larysz K, 2023 | 1 | 1 | 1 | 1 | 0 | 0 | 1 | 1 | 0 | 1 | 1 | 8 |
| Nowak A, 2023 | 1 | 1 | 1 | 0 | 0 | 1 | 0 | 1 | 0 | 1 | 0 | 6 |
| Rajbanshi I, 2023 | 1 | 1 | 1 | 0 | 0 | 1 | 1 | 1 | 0 | 1 | 0 | 7 |
| Shan C, 2022 | 1 | 1 | 1 | 0 | 0 | 1 | 1 | 1 | 1 | 1 | 0 | 8 |
| Bindayel IA, 2021 | 1 | 1 | 1 | 0 | 0 | 1 | 0 | 1 | 0 | 1 | 0 | 6 |
| Li Y, 2021 | 1 | 1 | 1 | 0 | 0 | 1 | 0 | 1 | 0 | 1 | 0 | 6 |
| Wang L, 2020 | 1 | 1 | 1 | 1 | 0 | 1 | 1 | 1 | 0 | 1 | 0 | 8 |
| Krul-Poel YHM, 2018 | 1 | 1 | 1 | 0 | 0 | 1 | 1 | 1 | 0 | 1 | 0 | 7 |
| Mogili KD, 2018 | 1 | 1 | 1 | 1 | 0 | 1 | 1 | 1 | 0 | 1 | 0 | 8 |
| Kumar A, 2017 | 1 | 1 | 1 | 1 | 0 | 1 | 0 | 1 | 0 | 0 | 0 | 6 |
| Scott D, 2016 | 1 | 1 | 1 | 0 | 0 | 1 | 1 | 1 | 0 | 1 | 0 | 7 |
| Moini A, 2015 | 1 | 1 | 1 | 1 | 0 | 1 | 0 | 1 | 0 | 1 | 0 | 7 |
| Bhattacharya SM, 2013 | 1 | 1 | 1 | 0 | 0 | 0 | 0 | 1 | 0 | 1 | 0 | 5 |
| Muscogiuri G, 2012 | 1 | 1 | 1 | 1 | 0 | 1 | 0 | 1 | 0 | 1 | 0 | 7 |
| Tsakova AD, 2012 | 1 | 1 | 0 | 0 | 0 | 1 | 0 | 1 | 0 | 1 | 0 | 5 |
| Li HWR, 2011 | 1 | 1 | 1 | 1 | 0 | 1 | 1 | 1 | 1 | 1 | 0 | 9 |
| Wehr E, 2011 | 1 | 1 | 1 | 0 | 0 | 1 | 0 | 1 | 0 | 1 | 0 | 6 |
| Wehr E, 2009 | 1 | 1 | 0 | 0 | 0 | 1 | 0 | 1 | 0 | 1 | 0 | 5 |
| Yildizhan R, 2009 | 1 | 1 | 1 | 1 | 0 | 1 | 0 | 1 | 0 | 1 | 0 | 7 |

Note: ① Whether the data source (survey, literature review) was clearly specified? ② Whether the inclusion and exclusion criteria for the two groups were listed or previous publications were referenced? ③ Whether the time period for patient identification was provided? ④ If not population-based, whether the study subjects were consecutive? ⑤ Whether the evaluator's subjective factors masked other characteristics of the study subjects? ⑥ Any quality assurance assessments were described (e.g., test/retest of the primary outcome measures); ⑦ The reasons for excluding any patients from the analysis were explained; ⑧ Measures for evaluating and/or controlling confounding factors were described; ⑨ If applicable, how missing data were handled in the analysis was explained; ⑩ The patient response rate and the completeness of data collection were summarized; ⑪ If follow-up was conducted, the expected percentage of incomplete patient data or the follow-up results were identified.

# Supplementary Table 4: Quality Assessment of Case-control Studies

| **Included Studies** | **Selection of Case and Control Groups** | | | | **Comparability** | **Exposure Factors** | | | **Total Score** |
| --- | --- | --- | --- | --- | --- | --- | --- | --- | --- |
|  | **Appropriateness of Case Definition and Diagnosis** | **Case Representativeness** | **Control Selection** | **Control Definition** | **Comparability of Cases and Controls** | **Investigation and Assessment of Exposure** | **Methods for Cases and Controls** | **Non-response Rate** |  |
| Yari F, 2025 | 1 | 1 | 0 | 1 | 2 | 1 | 1 | 0 | 7 |
| Chakraborty S, 2025 | 1 | 1 | 1 | 1 | 1 | 1 | 1 | 0 | 7 |
| Akinola LA, 2024 | 1 | 1 | 0 | 1 | 0 | 1 | 1 | 0 | 5 |
| Davis EM, 2019 | 1 | 1 | 0 | 1 | 2 | 1 | 1 | 0 | 7 |
| Kensara OA, 2018 | 1 | 1 | 1 | 1 | 2 | 1 | 1 | 0 | 8 |
| Ng BK, 2017 | 1 | 1 | 0 | 1 | 0 | 1 | 1 | 1 | 6 |
| Ganie MA, 2016 | 1 | 1 | 1 | 1 | 1 | 1 | 1 | 0 | 7 |
| Figurová J, 2015 | 1 | 1 | 1 | 1 | 1 | 1 | 1 | 0 | 7 |
| Sadhir M, 2015 | 1 | 1 | 0 | 1 | 2 | 1 | 1 | 0 | 7 |
| Kim JJ, 2014 | 1 | 1 | 0 | 1 | 2 | 1 | 1 | 0 | 7 |

# Supplementary Figure 1: Forest plot of overall prevalence


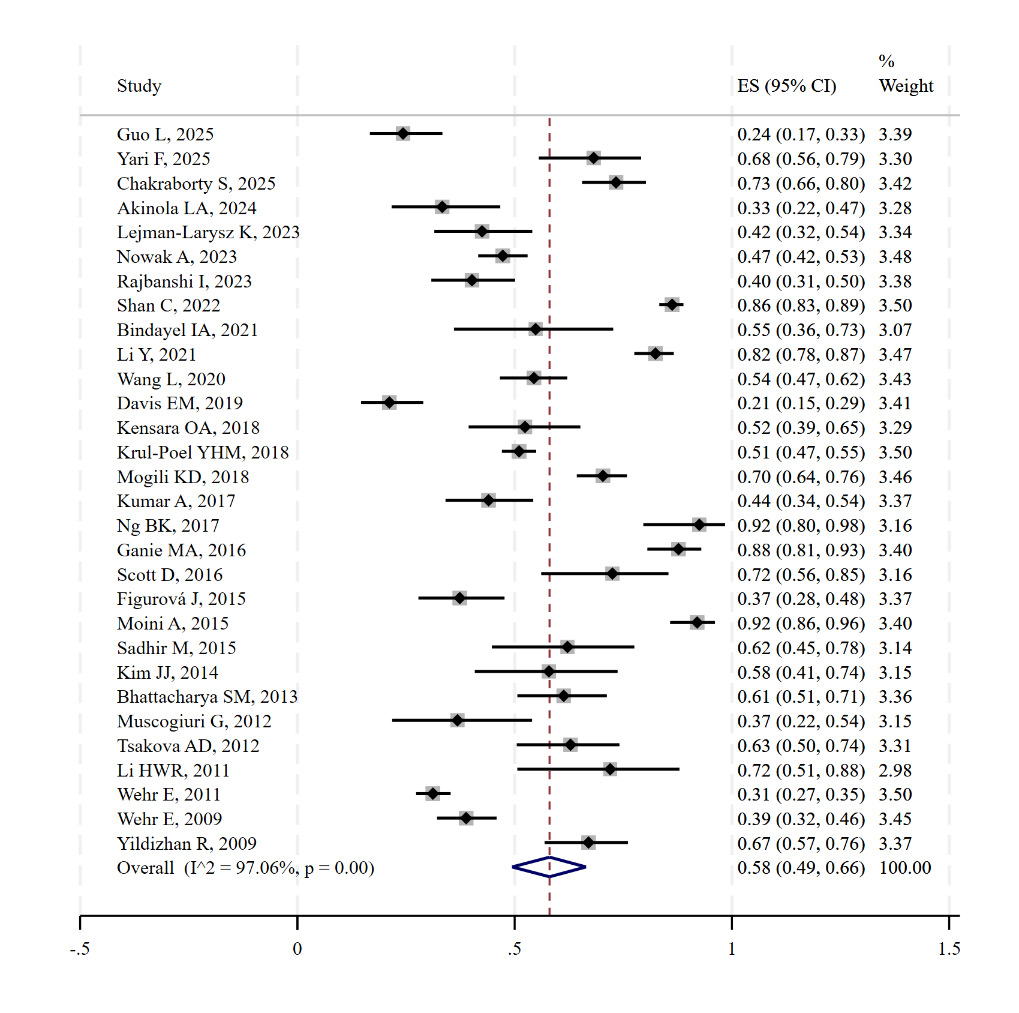


# Supplementary Figure 2: Forest plot of subgroup analysis by study type


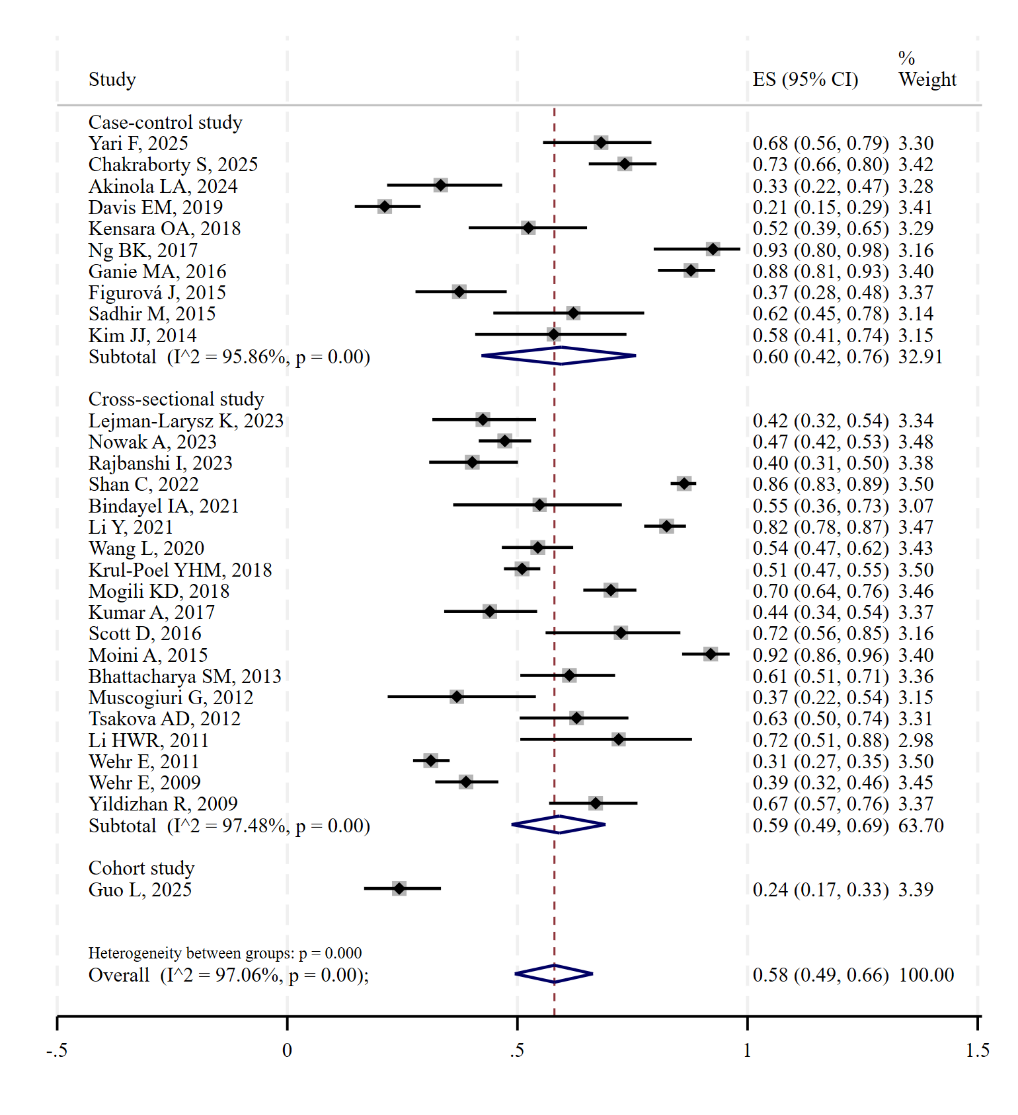


# Supplementary Figure 3: Forest plot of subgroup analysis by research region


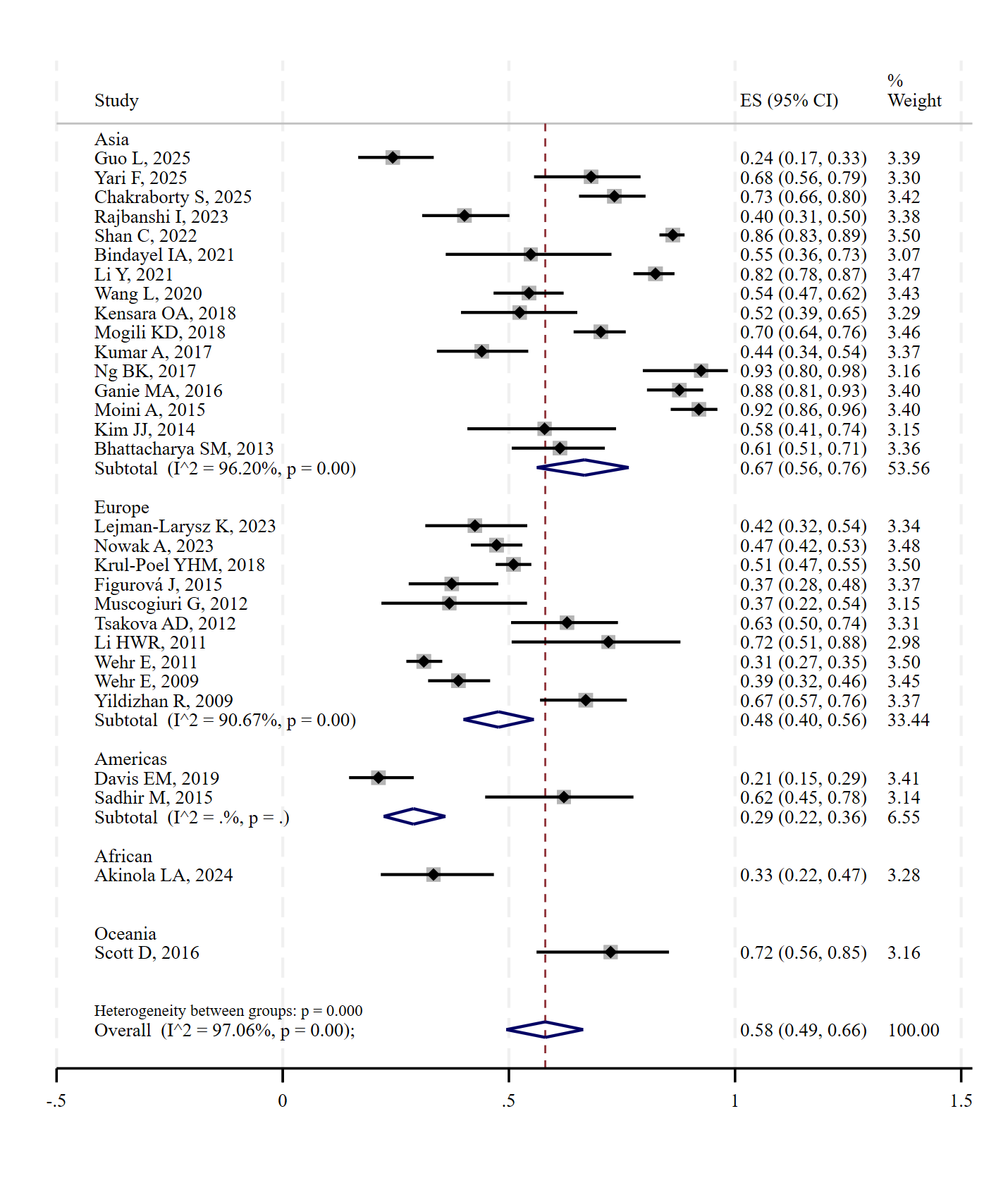


# Supplementary Figure 4: Forest plot of subgroup analysis by national development status


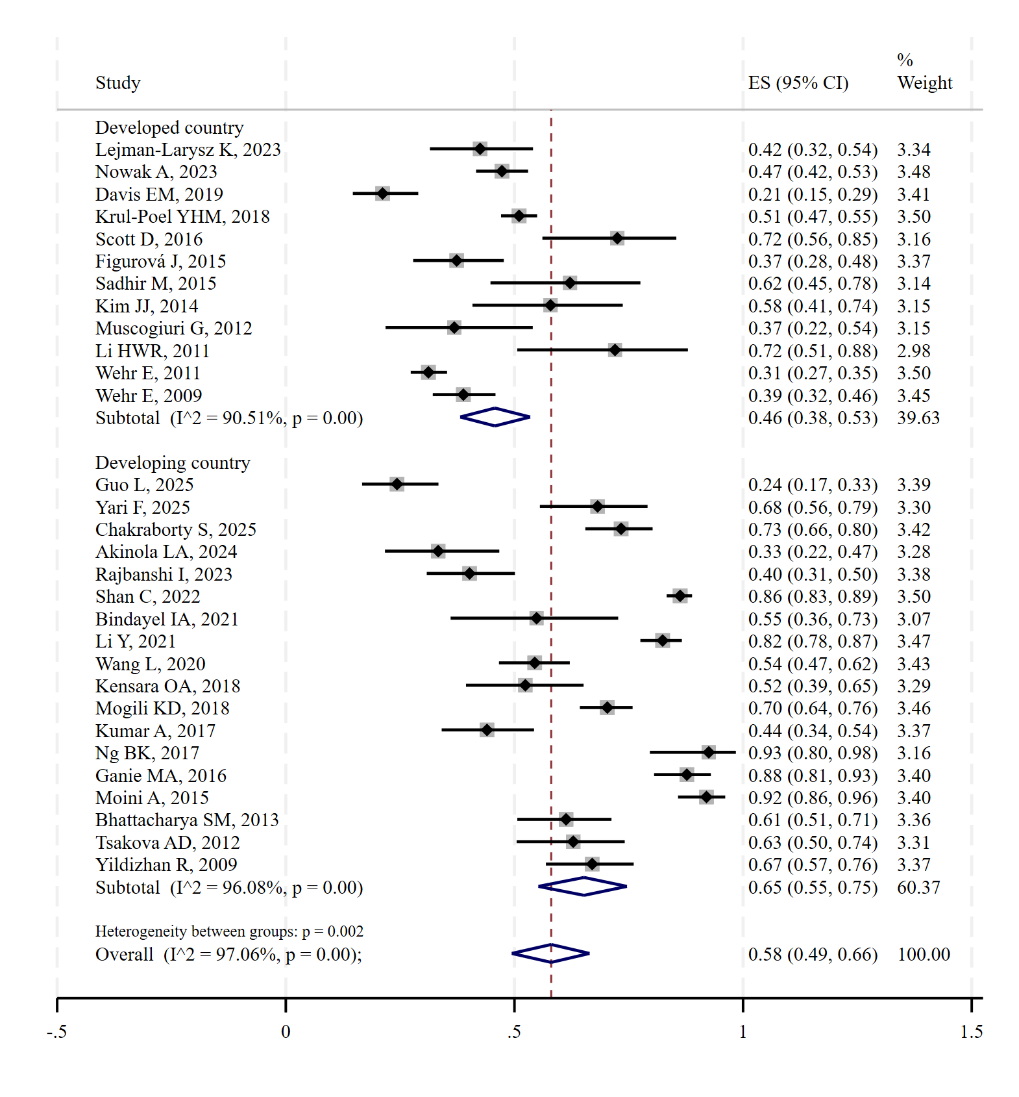


# Supplementary Figure 5: Forest plot of subgroup analysis by diagnostic criteria for PCOS


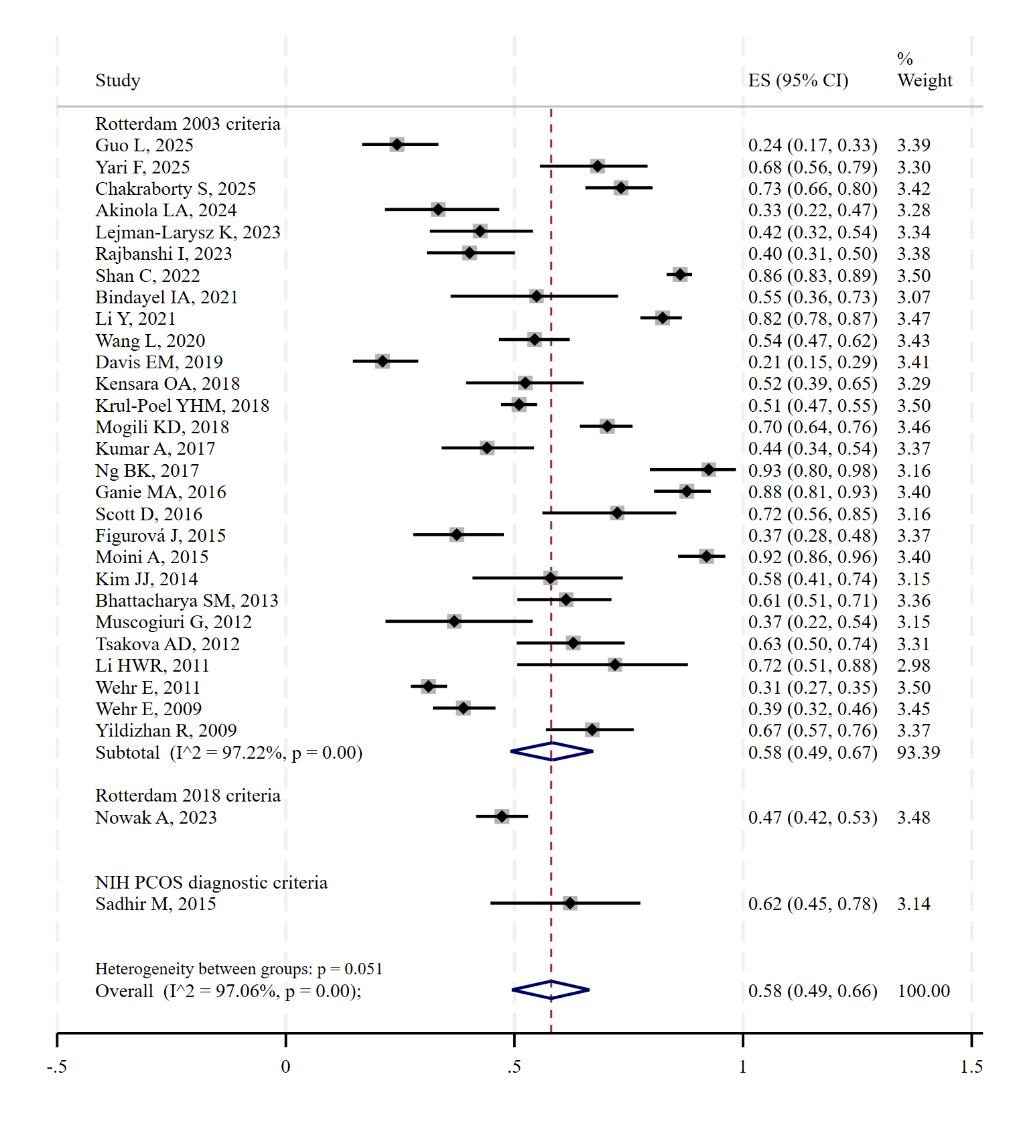


# Supplementary Figure 6: Forest plot of subgroup analysis by 25(OH)D measurement method


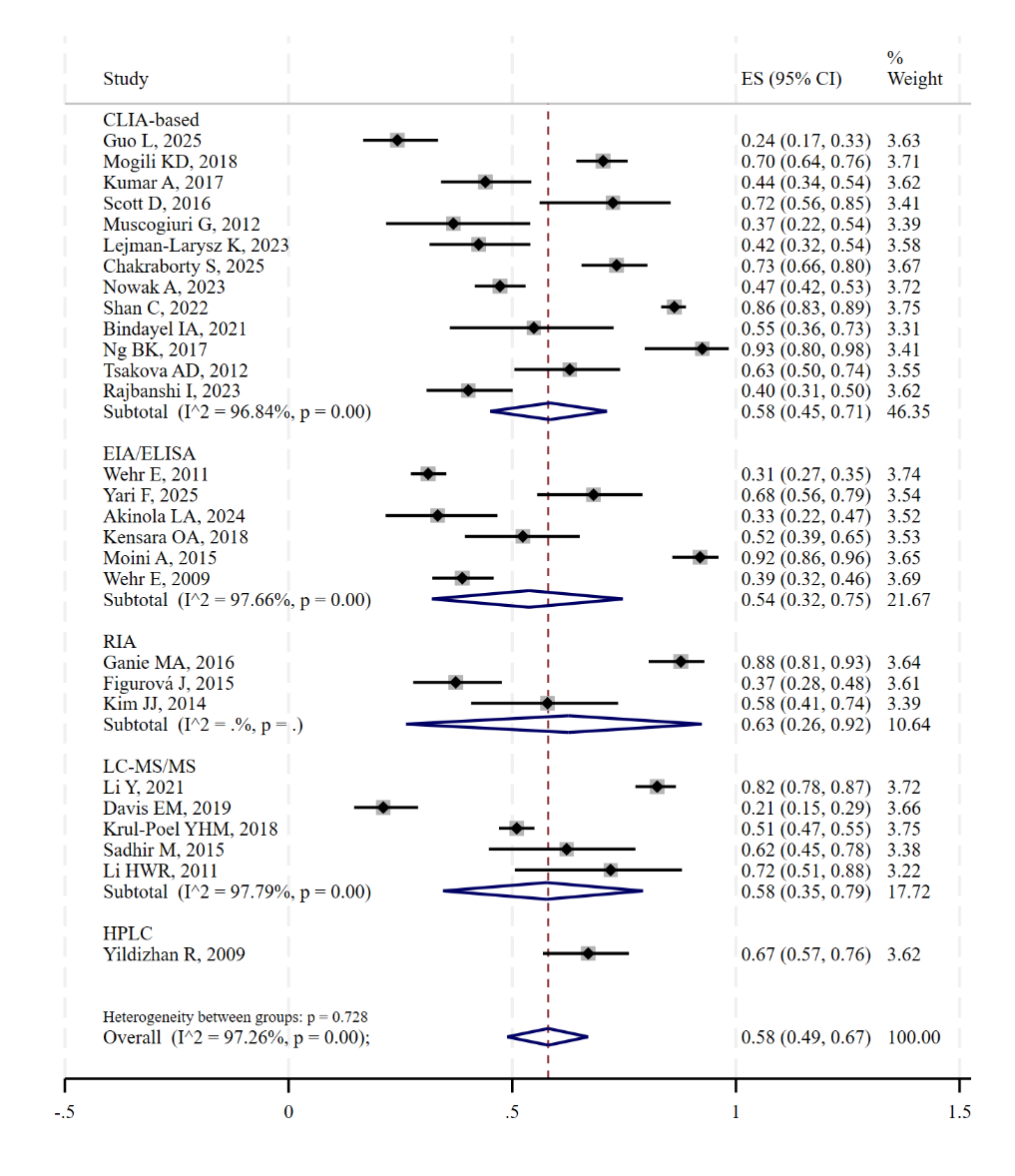


# Supplementary Figure 7: Forest plot of subgroup analysis by mean age


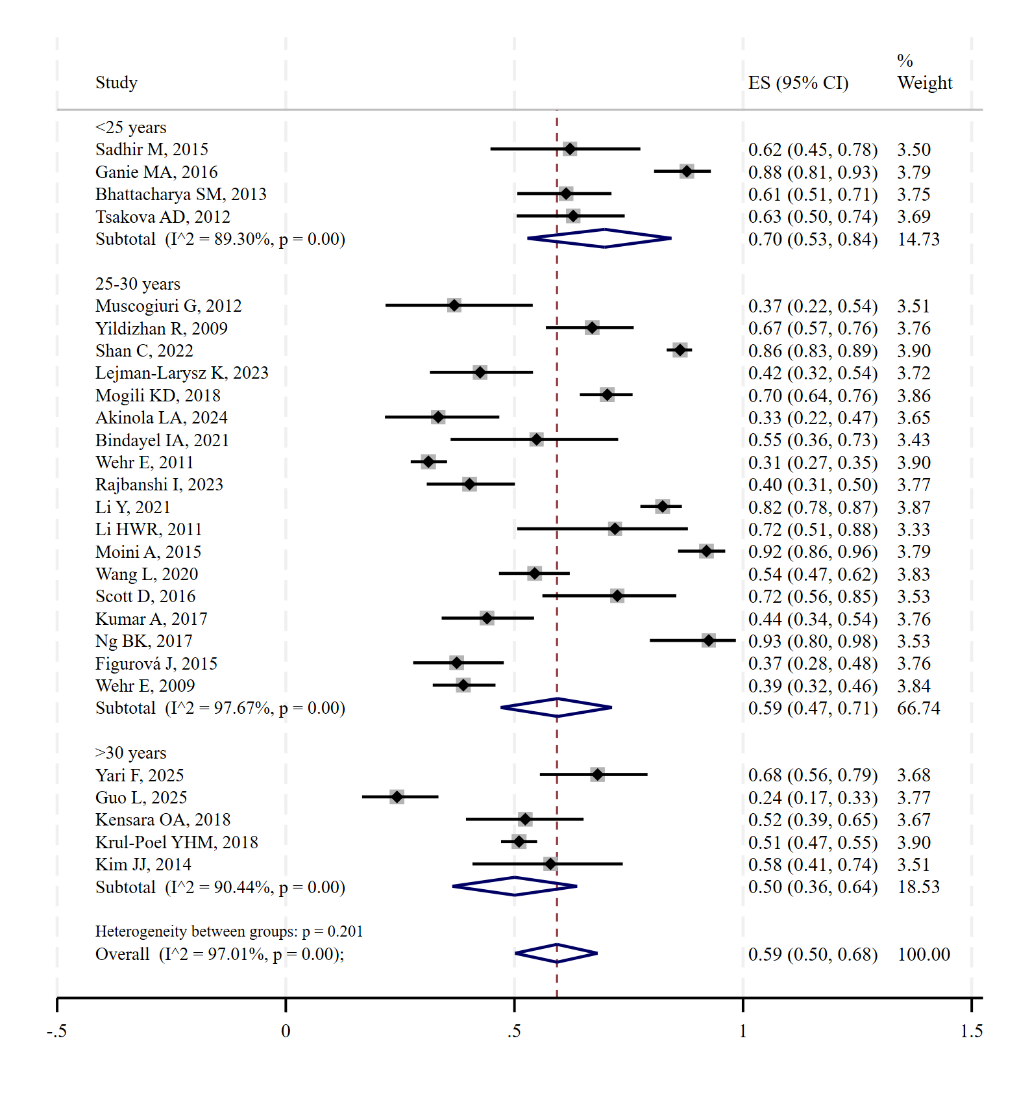


# Supplementary Figure 8: Forest plot of subgroup analysis by mean BMI


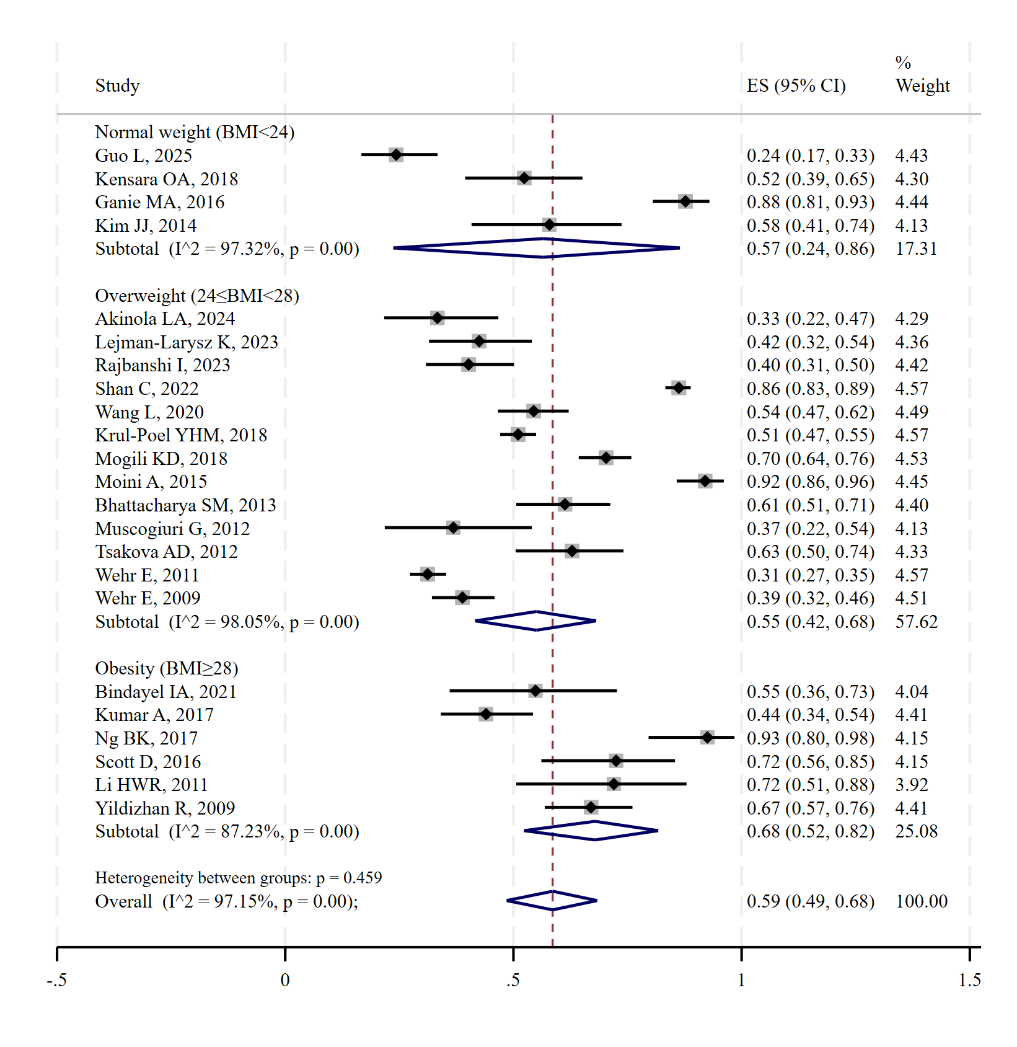


# Supplementary Figure 9: Forest plot of subgroup analysis by sample size


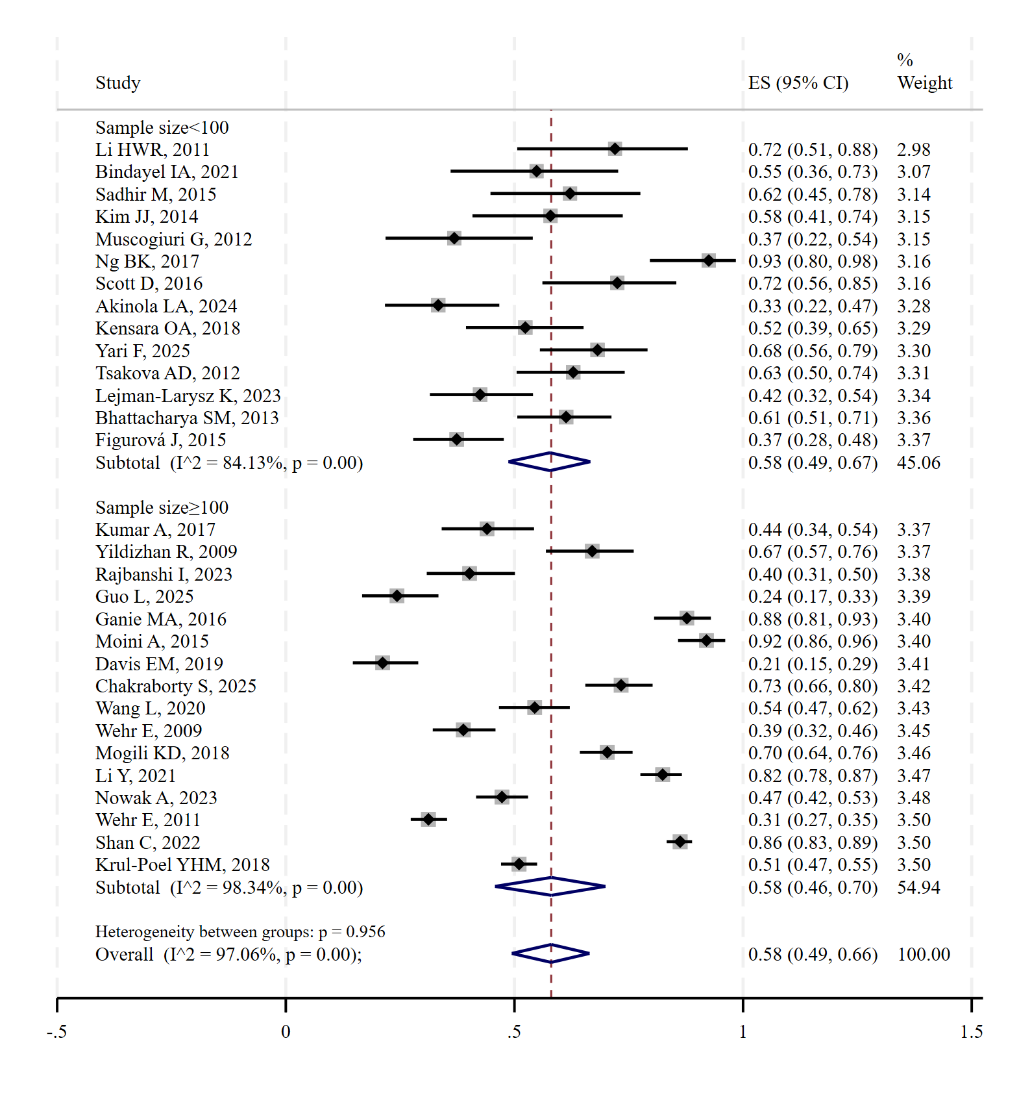


# Supplementary Figure 10: Meta-regression plot by study type


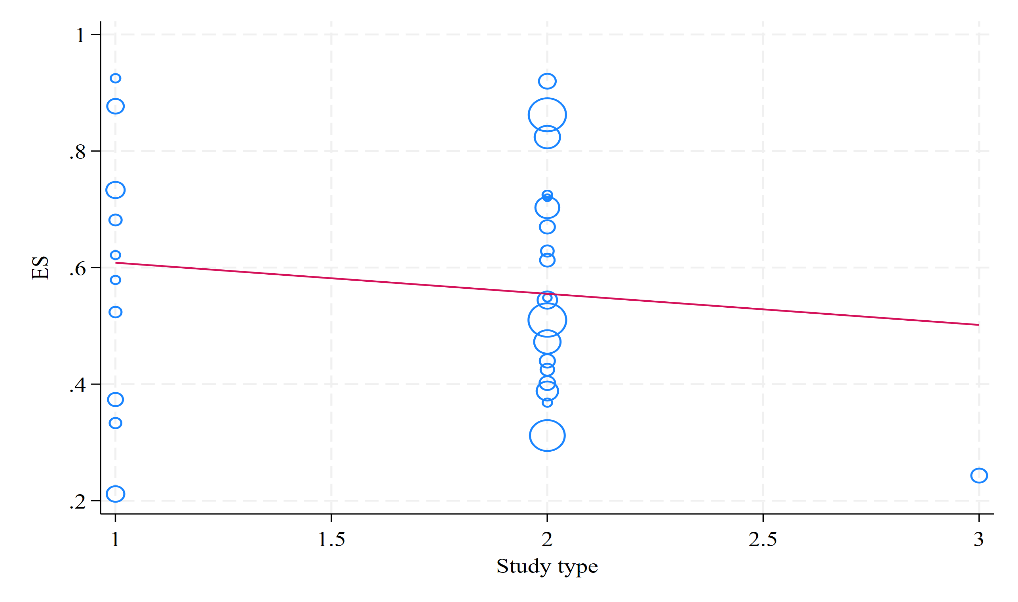


# Supplementary Figure 11: Meta-regression plot by research region


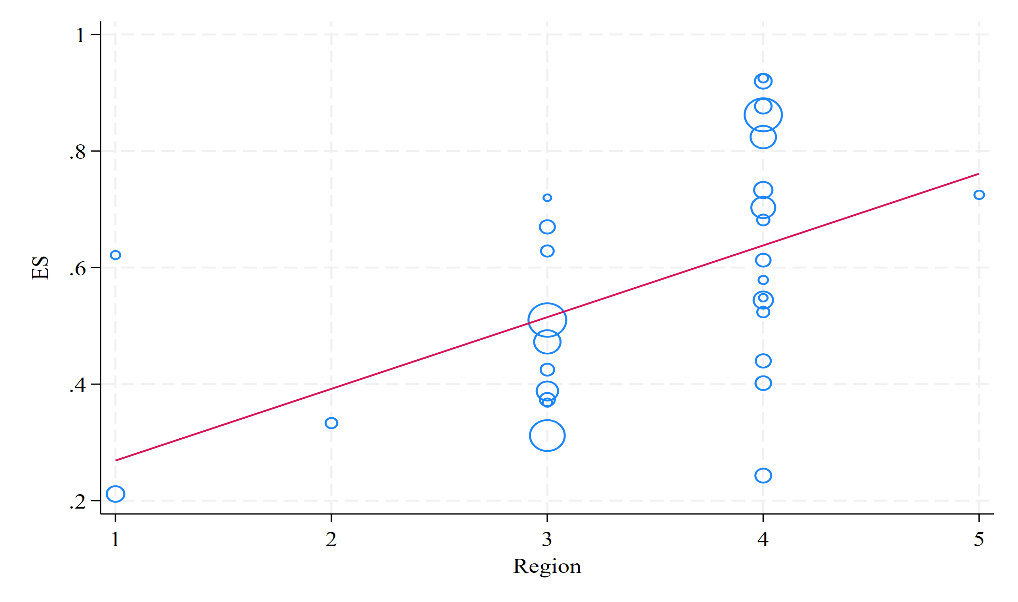


# Supplementary Figure 12: Meta-regression plot by national development status


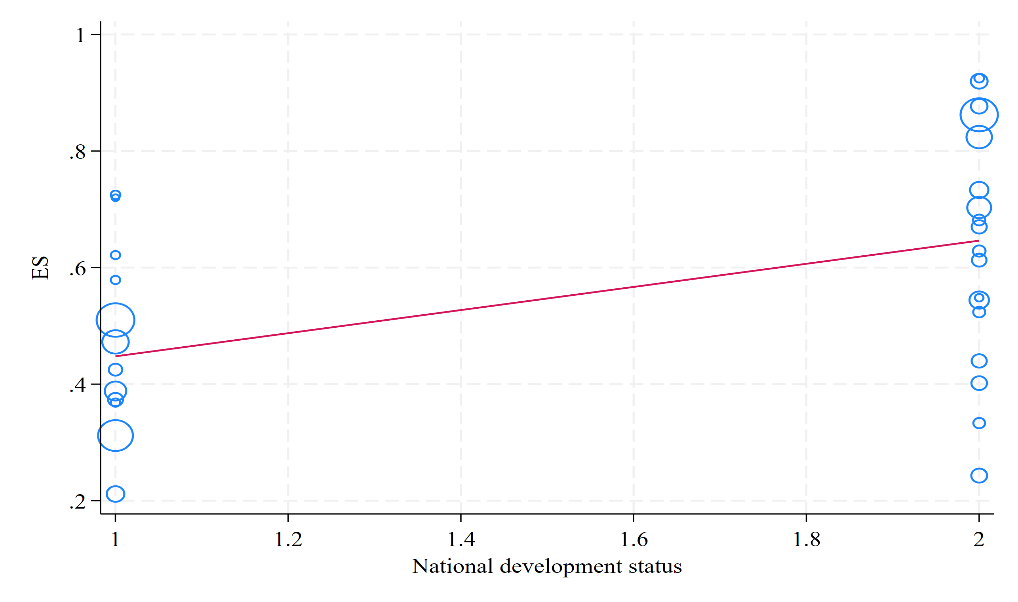


# Supplementary Figure 13: Meta-regression plot by diagnostic criteria for PCOS


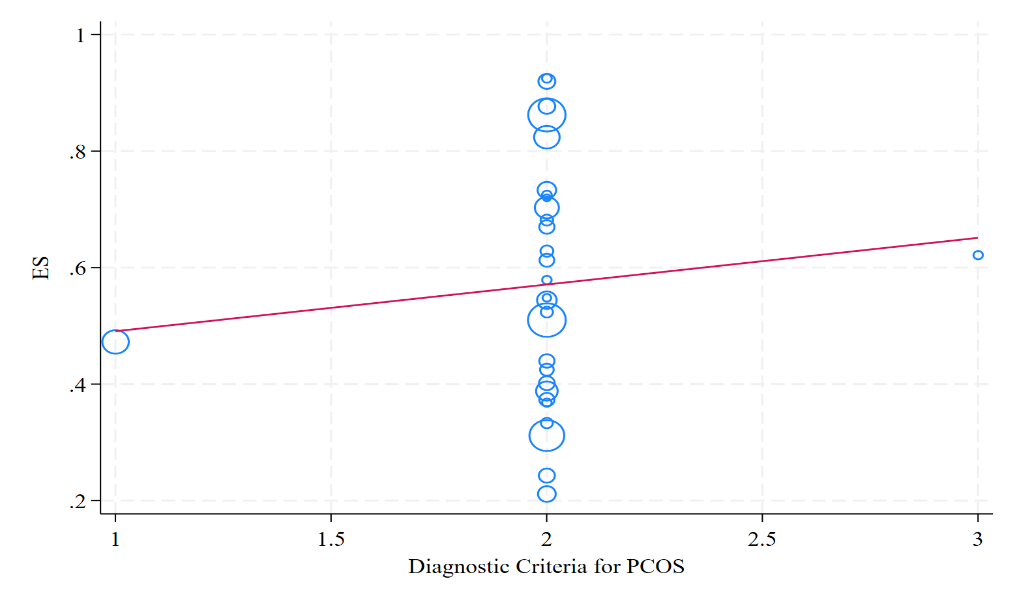


# Supplementary Figure 14: Meta-regression plot by 25(OH)D measurement method


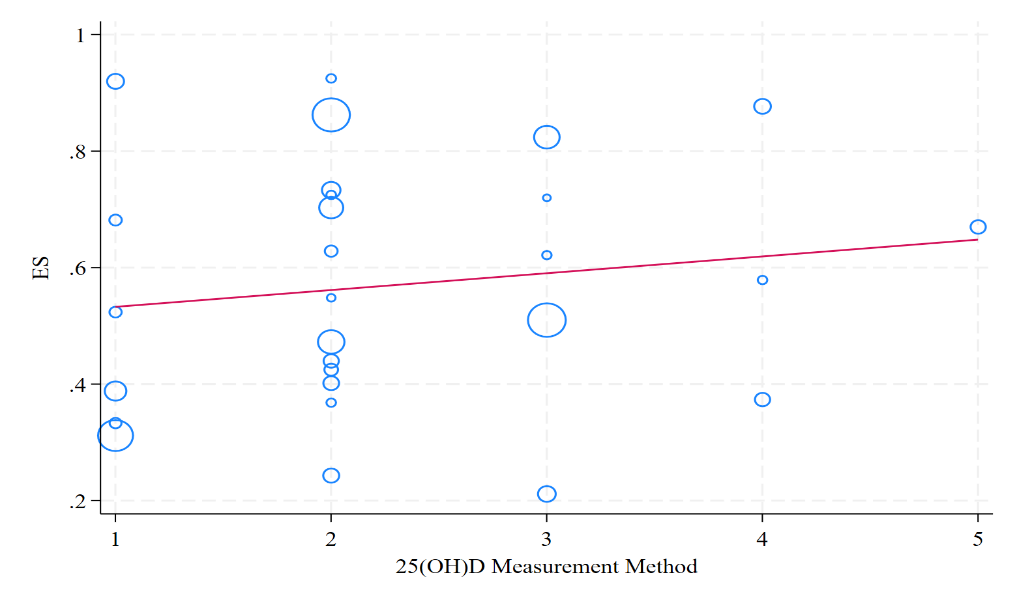


# Supplementary Figure 15: Meta-regression plot by mean age


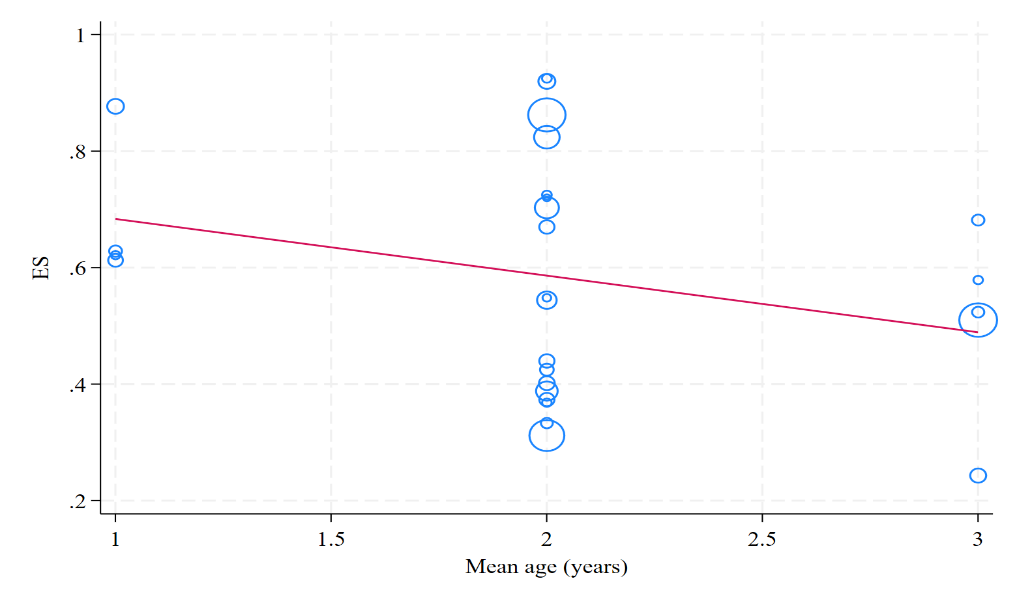


# Supplementary Figure 16: Meta-regression plot by mean BMI


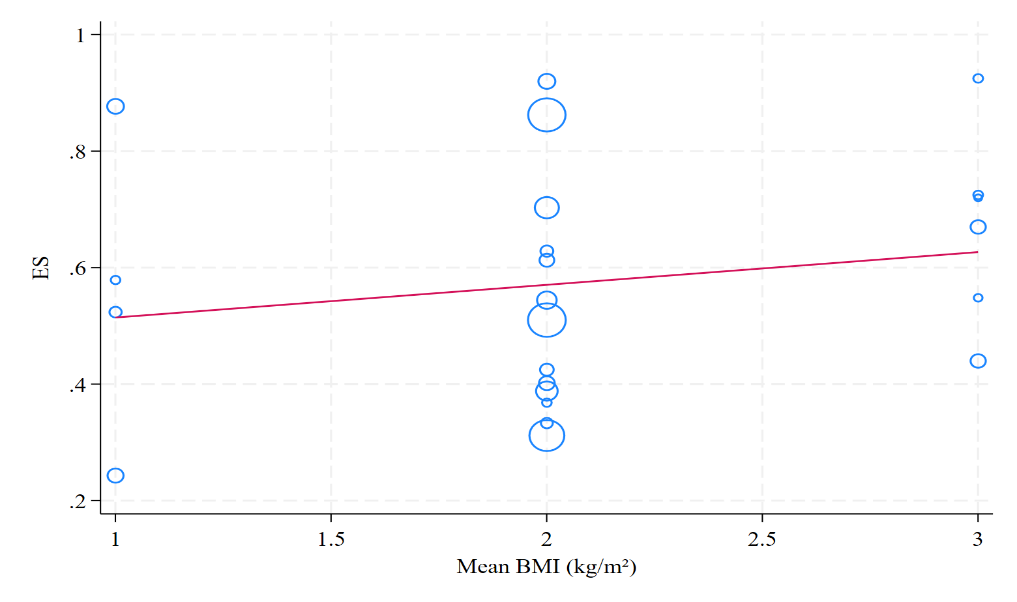


# Supplementary Figure 17: Meta-regression plot by sample size


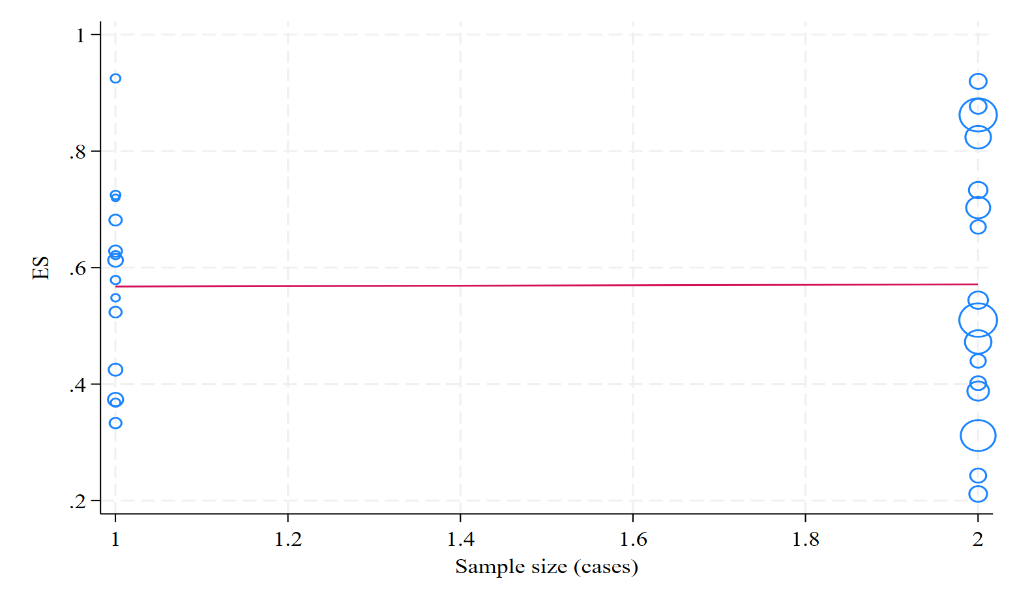


# Supplementary Figure 18: Sensitivity analysis plot


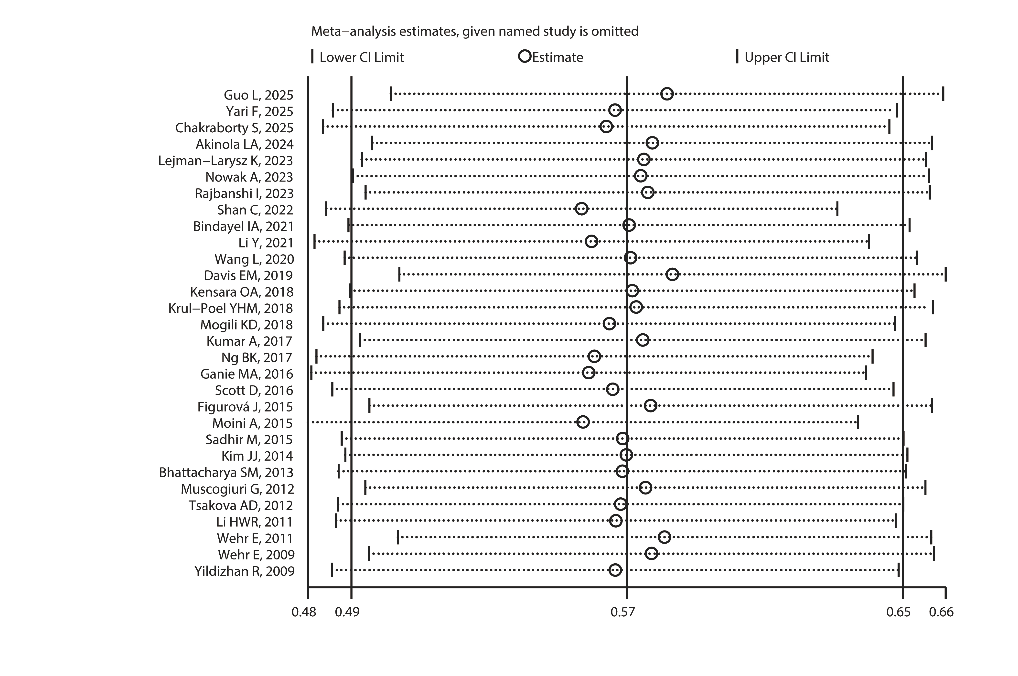


# Supplementary Figure 19: Publication bias funnel plot


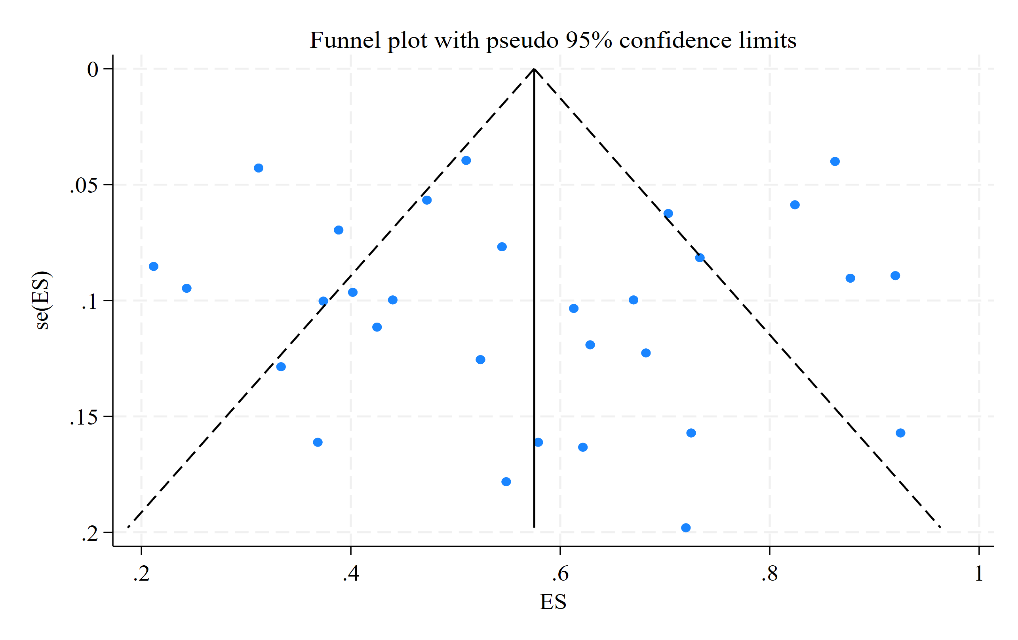


# Supplementary Figure 20: Funnel plot of trim-and-fill method


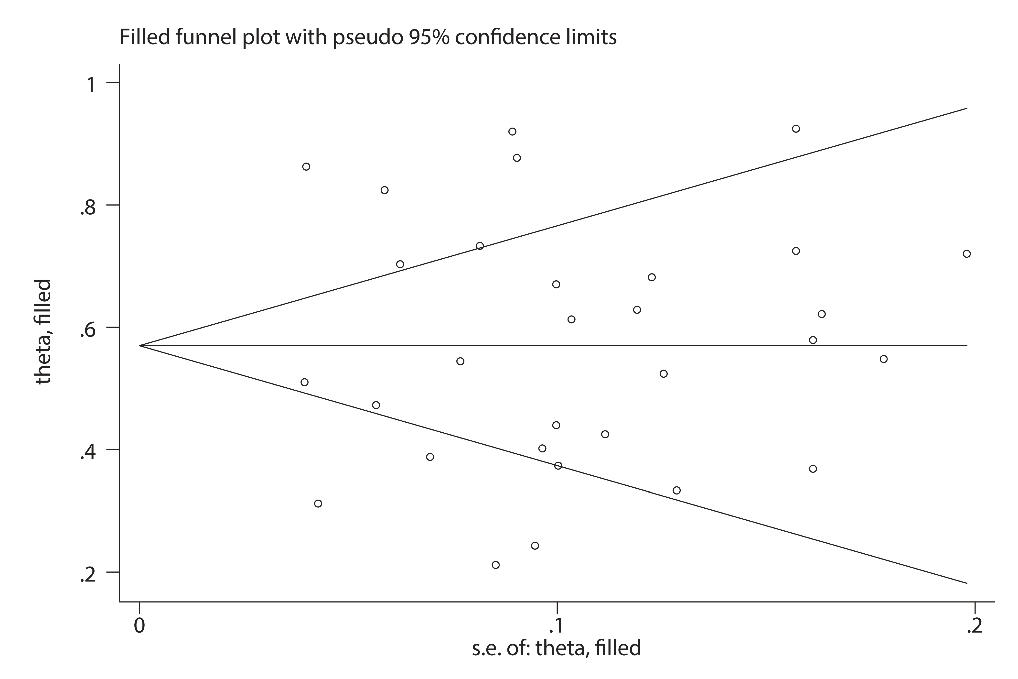

Supplement: Supplementary file 1 [file Supplementary_file_1.DOCX]
